# Combined gene essentiality scoring improves the prediction of cancer dependency maps

**DOI:** 10.1016/j.ebiom.2019.10.051

**Published:** 2019-11-12

**Authors:** Wenyu Wang, Alina Malyutina, Alberto Pessia, Jani Saarela, Caroline A. Heckman, Jing Tang

**Affiliations:** a Research Program in Systems Oncology, Faculty of Medicine, University of Helsinki, Haartmaninkatu 8, FI-00014 Helsinki, Finland; b Institute for Molecular Medicine Finland (FIMM), University of Helsinki, Tukholmankatu 8, FI-00014 Helsinki, Finland

**Keywords:** Functional genetic screen, CRISPR, RNAi, Gene essentiality, Data integration

## Abstract

**Background:**

Probing genetic dependencies of cancer cells can improve our understanding of tumour development and progression, as well as identify potential drug targets. CRISPR-Cas9-based and shRNA-based genetic screening are commonly utilized to identify essential genes that affect cancer growth. However, systematic methods leveraging these genetic screening techniques to derive consensus cancer dependency maps for individual cancer cell lines are lacking.

**Finding:**

In this work, we first explored the CRISPR-Cas9 and shRNA gene essentiality profiles in 42 cancer cell lines representing 10 cancer types. We observed limited consistency between the essentiality profiles of these two screens at the genome scale. To improve consensus on the cancer dependence map, we developed a computational model called combined essentiality score (CES) to integrate the genetic essentiality profiles from CRISPR-Cas9 and shRNA screens, while accounting for the molecular features of the genes. We found that the CES method outperformed the existing gene essentiality scoring approaches in terms of ability to detect cancer essential genes. We further demonstrated the power of the CES method in adjusting for screen-specific biases and predicting genetic dependencies in individual cancer cell lines.

**Interpretation:**

Systematic comparison of the CRISPR-Cas9 and shRNA gene essentiality profiles showed the limitation of relying on a single technique to identify cancer essential genes. The CES method provides an integrated framework to leverage both genetic screening techniques as well as molecular feature data to determine gene essentiality more accurately for cancer cells.

## 1 Introduction

Interrogating the genetic dependencies of cancer cells provides important evidence for target-based drug discovery [1]. Loss-of-function screens have emerged as powerful tools to introduce genetic perturbations *in vitro*, providing new opportunities to identify genes that are essential for cell survival and proliferation [2]. To carry out a systematic exploration of genome-scale cancer dependency profiles, these genetic screens rely on a library containing various synthetized short sequence constructs designed to target specific genes. Using an optimized delivery system, the library as a whole can be efficiently introduced into a cell culture, resulting in a mixture of cell subpopulations, each carrying one sequence construct that triggers the depletion of a particular gene. During the culture period, the cell subpopulations depleted of essential genes will lose fitness, resulting in under-representation of their effector sequence constructs [3]. To quantify the degree of essentiality for individual genes (i.e. the gene essentiality score), genomic DNA is isolated from the cell culture both at the initial condition and at the end of the culture period. Using PCR and next-generation sequencing technologies, depletion of corresponding sequence constructs can be determined subsequently.

Over the last decade, short hairpin RNA (shRNA), together with the more recently developed CRISPR-Cas9-based sgRNA (single guide RNA) have been adopted as two major techniques to conduct genome-scale loss-of-function screens. The shRNA-based and CRISPR-based screens involve construction of synthetic oligonucleotide sequences that are delivered into cells to activate distinct loss-of-function machineries: shRNA is directed to bind to its target mRNA in the cytoplasm via the RNAi (RNA interference) pathway, leading to degradation of the target mRNA and loss of gene expression without altering the genome of the cells (i.e. a transient

**Research in context**

**Evidence before this study**

Interrogating the genetic dependencies of cancer cells provides important evidences for target-based drug discovery. RNAi-based shRNA and CRISPR-Cas9-based sgRNA have been commonly utilized in functional genetic screens to derive cancer dependence maps. However, previous studies suggested limited overlap of essentiality profiles based on the two technologies. Existing computational methods mainly focused on estimation of true gene essentiality from genetic screens using single technologies, but integrative methods to combine the gene essentiality profiles from both CRISPR and shRNA screens are lacking.

**Added value of this study**

In the current study, we developed a computational approach called combined gene essentiality score (CES) to integrate CRISPR and shRNA gene essentiality profiles and the molecular features of cancer cells. We showed that CES significantly improved the performance of gene essentiality prediction for shared genetic dependencies across multiple cell lines as well as for therapeutic targets that are selective for a specific cancer cell line.

**Implications of all the available evidence**

The CES approach provides an effective data integration strategy to allow improved estimation of cancer dependency maps, which may facilitate the discovery of therapeutic targets for personalized medicine. Although we have focused on the genetic screens that are largely restricted for the cell growth phenotype, the CES modelling strategy itself is applicable to interrogate genes that are essential for other image-based or antibody-based phenotypes, thus further accelerating the translation from biomedical discoveries to novel therapeutic development.

**CRediT authorship contribution statement**

**Wenyu Wang:** Conceptualization, Data curation, Formal analysis, Visualization, Writing - original draft, Writing - review & editing. **Alina Malyutina:** Data curation, Writing - review & editing. **Alberto Pessia:** Conceptualization, Methodology, Writing - review & editing. **Jani Saarela:** Writing - review & editing. **Caroline A. Heckman:** Writing - review & editing. **Jing Tang:** Conceptualization, Formal analysis, Funding acquisition, Methodology, Project administration, Supervision, Writing - original draft, Writing - review & editing.

Alt-text: Unlabelled Box

knockdown effect). In contrast, sgRNA utilizes the CRISPR pathway to direct the Cas9 protein to cut genomic DNA in the nucleus, triggering the non-homologous end joining (NHEJ) pathway to introduce permanent loss-of-function mutations, which result in complete and permanent knockout of the target genes [2], [4], [5]. Despite the relative simplicity in experimental setups, the efficiency and specificity of shRNA and sgRNA constructs need to be optimized for reliable detection of cancer essential genes. For example, evidence suggests that both shRNAs and sgRNAs may affect additional off-target genes due to partial sequence complementarity, and therefore introduce experimental noise that masks the actual cellular response to the intended gene depletions [1], [6]. Differences in gene-depletion efficiency may also contribute to the experimental variability in shRNA screens [7] as well as CRISPR-Cas9 screens [8]. To optimize the design of the sequence library, computational methods have been developed to predict the on-target efficiency and off-target activity of shRNA or sgRNA sequences [8], [9], [10].

To further improve the accuracy of functional genetic screens, another class of computational methods has focused on the estimation of true gene essentiality from noisy experimental results, while accounting for confounding factors. For example, recent publications have reported that increased genomic amplification and *TP53* mutation status may confound the gene essentiality estimates in CRISPR screens [11], [12], [13], [14]. A computational method called CERES has been developed to adjust for the inflated essentiality scores of genes in genomic amplification regions [11]. On the other hand, computational methods including DEMETER [15] have been proposed to adjust the off-target effects mediated by micro-RNA pathways, which are known to be more prominent in shRNA screens than in CRISPR screens.

With the increasing maturity and wide application of both CRISPR and shRNA screening technologies, attempts have been made to integrate their gene essentiality profiles in order to derive a more unbiased cancer dependence map [16], [17], [18]. However, it is reported that the identified essential genes from the two techniques overlapped only partially. Two recent studies carried out CRISPR and shRNA screens in parallel for several human cancer cell lines [4], [19], with different conclusions being made in terms of the accuracy for detecting truly essential genes. For example, Evers et al. reported a superior prediction accuracy with CRISPR screens compared to shRNA screens [19], whereas Morgens et al. observed a similar level of prediction performance [4]. However, Morgens et al. showed that a large proportion of essential genes identified by CRISPR screens were not replicated in shRNA screens and vice versa, suggesting the presence of complex confounding factors that are inherently distinct between these two technologies. Moreover, these comparative studies were conducted on a few genes and cell lines; therefore, it remains unclear whether their conclusions can be generalized. For example, Evers et al. investigated the essentiality profiles for a set of 46 essential and 47 non-essential genes in two cancer cell lines (RT-112 and UM-UC-3), whereas Morgens et al. analysed a larger gene set including 217 essential and 947 non-essential genes, but the comparison was made using only one cell line, K562.

In this study, we carried out a systematic comparison for CRISPR- and shRNA-based gene essentiality profiles across a larger collection of cancer cell lines. We found that the CRISPR and shRNA-based gene essentiality profiles showed limited consistency at the genome-wide level. To improve the estimation of true essentiality, we developed a computational approach called combined gene essentiality score (CES) to integrate CRISPR and shRNA gene essentiality profiles as well as the molecular features of cancer cells. We showed that CES significantly improved the performance of gene essentiality prediction for shared genetic dependencies across multiple cell lines as well as for therapeutic targets that are selective for a specific cancer cell line. The CES approach thus provides an effective data integration strategy to allow improved estimation of cancer dependency maps, which may facilitate the discovery of therapeutic targets for personalized medicine. The source code to replicate this analysis is available at https://github.com/Wenyu1024/CES.

## 2 Materials and methods

### 2.1 Data collection

**Fig. 1:**
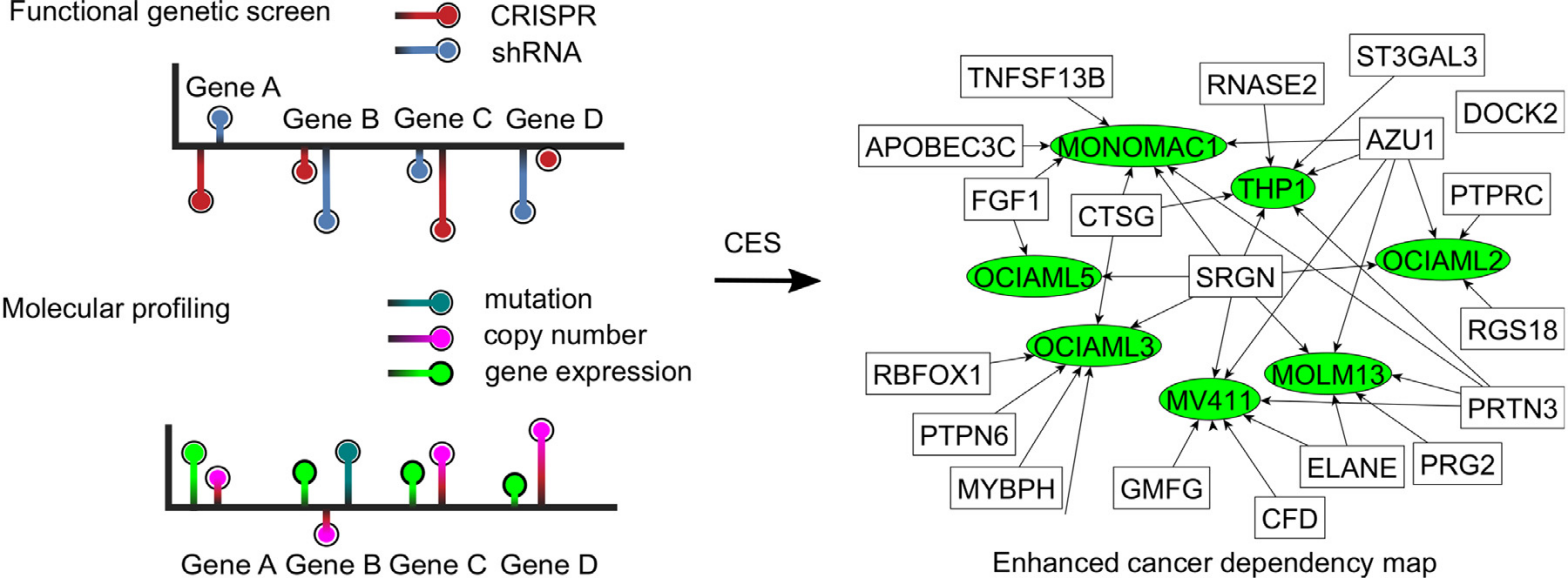
The CES data integration pipeline to improve identification of cancer essential genes based on functional genetic screens and molecular feature data. Genome-wide shRNA and CRISPR-Cas9 based essentiality scores as well as molecular profiles for each cell line were obtained from public databases and literature. For a gene in a given cell line, a feature vector was constructed including CRISPR-based essentiality scores, shRNA-based essentiality scores, as well as mutation count, RPKM from RNA-seq, mRNA expression from microarray, and copy number variation. The aim of CES is to provide a data integration model to improve the consensus estimation of essential genes in cancer.

A total of 42 cancer cell lines with both CRISPR and shRNA screenings performed at the genome-scale were included for the study. CRISPR-based gene essentiality scores were obtained from the Achilles study (v3.38) [12] and three other studies [20], [21], [22]. CRISPR-based gene essentiality scores were determined from their corresponding level essentiality depletion scores using different strategies. For example, the Achilles study used the second-top essential sgRNA depletion score to represent the CRISPR-based gene essentiality, whereas the other studies utilized either arithmetic averaging [21] or a Bayesian modelling averaging strategy [20], [22], [23]. On the other hand, shRNA-based gene essentiality scores were obtained by arithmetic averaging over multiple shRNA-level depletion scores from the Achilles study (v2.20) [15]. Molecular features for these cell lines including mutation, gene expression, and copy number variation were obtained from the Cancer Cell Line Encyclopaedia (CCLE) database [24]. More specifically, point mutations and indels were captured by targeted massively parallel sequencing and were transformed into mutation counts for individual genes. Gene expression features were represented via the RNA-Seq-based RPKM counts and Affymetrix array-based log2 intensity values, whereas the normalized log2 ratios of CN/2 from Affymetrix SNP array were utilized as copy number variations. The resulting data matrix thus contained the shRNA-based and CRISPR-based essentiality scores, as well as the molecular profiles for each gene in a given cell line (Fig. 1). All the features were normalized as z-scores at each cell line for further analyses, resulting in a data matrix with 16,492 genes for a total of 42 cell lines. A detailed list of the data sources can be found in Supplementary Table 1.

### 2.2 Comparison of shRNA and CRISPR-based essentiality scores

We first ran a genome-wide comparison between shRNA- and CRISPR-based essentiality scores on the 42 cancer cell lines. We followed the convention that has been adopted in major computational methods including DEMETER and CERES, where a lower and more negative essentiality score results from greater depletion of cancer cells upon genetic perturbation and thus represents higher essentiality. Pearson correlation was employed to investigate the consistency between shRNA and CRISPR scores, where a higher correlation indicated better between-screen consistency whereas a zero or negative correlation indicated poor between-screen consistency. We also used the mean squared error (MSE) between shRNA and CRISPR essentiality scores to evaluate their consistency.

### 2.3 The CES model to integrate functional screen and molecular feature data

We proposed a combined essentiality score (CES) that integrates CRISPR and shRNA-based gene essentiality scores and molecular features. Specifically, for a gene i,i=1,…,M in cell line j,j=1,…,N, the CES can be determined as

(1) $$\begin{array}{ccc} \text{CE}S_{ij} & = & \beta_{j}+\theta_{s}\text{shRN}A_{ij}+\theta_{m}\text{MU}T_{ij}+\theta_{c}\text{CN}V_{ij}\\ & & +\;\theta_{a}\text{EXP}.\text{arra}y_{ij}+\theta_{r}\text{EXP}.\text{RNAse}q_{ij}, \end{array}$$

where the parameters in Eq. (1) are determined by minimizing the following objective function:

(2) $$\sum_{i=1}^{M}\sum_{j=1}^{N}{\left(\text{CE}S_{ij}-\text{CRISP}R_{ij}\right)}^{2}$$

To solve the linear least squares problem, a QR decomposition algorithm is utilized [25].

The CES defined in Eq. (1) can be rewritten as a weighted average of CRISPR- and shRNA-based gene essentiality scores,

(3) $$\text{CE}S_{ij}=\left(1-\alpha_{ij}\right)\text{CRISP}R_{ij}\;+\;\alpha_{ij}\text{shRN}A_{ij\;},$$

where the weight *α_ij_* is affected by the molecular features as

(4) $$\alpha_{ij}=\frac{\text{CE}S_{ij}-\text{CRISP}R_{ij}}{\text{shRN}A_{ij}-\text{CRISP}R_{ij}}=\frac{\beta_{j}+\theta_{s}\text{shRN}A_{ij}+\theta_{m}\;\text{MU}T_{ij}+\theta_{c}\text{CN}V_{ij}+\theta_{a}\text{Exp}.\text{arra}y_{ij}+\theta_{r}\text{EXP}.\text{RNAse}q_{ij}-\text{CRISP}R_{ij}}{\text{shRN}A_{ij}-\text{CRISP}R_{ij}}$$

Note that Eq. (3) does not imply that CES is a linear combination of CRISPR and shRNA scores; rather, their relationship is affected by molecular features that are gene- and cell-line-specific, which can be captured in the model.

### 2.4 Model comparison

We compared the CES model using three baseline models, including:

1) SA: a simple averaging model, where$\;\alpha_{ij}=0.5$, i.e.
  (5) $$SA_{ij}\;=\;\frac{\left(\text{CRISP}R_{ij}\;+\;\text{shRN}A_{ij}\right)}{2}$$
2) CES^null^: a CES model where the molecular signature information is removed from Eq. (1), i.e.
  (6) $${\text{CES}}_{ij}^{\text{null}}=\beta_{j}+\theta_{s}\;\text{shRN}A_{ij}$$
3) CES^perm^: a CES model in which the molecular signatures are randomly shuffled, i.e.
  (7) $$\begin{array}{ccc} {\text{CES}}_{ij}^{\text{perm}} & = & \beta_{j}+\theta_{s}\text{shRN}A_{ij}+\theta_{m}\text{MU}T_{i^{*}j^{*}}+\theta_{c}\text{CN}V_{i^{*}j^{*}}\\ & & +\;\theta_{a}\text{EXP}.\text{arra}y_{i^{*}j^{*}}\;+\theta_{r}\text{EXP}.\text{RNAse}q_{i^{*}j^{*}} \end{array}$$

where i*, j* refer to a gene and a cell line that are randomly selected to be different from (*i, j*) .

The SA model was considered as a baseline as it assigns equal weights to the CRISPR and shRNA-based screens, assuming that their gene essentiality profiles are generated from the same distribution. The CES^null^ and CES^perm^ models were used to evaluate the relevance of molecular features. Namely, if molecular signatures of a cell line play significant roles in defining true gene essentiality, then the CES model should perform better than the CES^null^ and CES^perm^ models that contain null or randomized molecular information.

Furthermore, we compared the CES model with the CERES model [11], DEMETER1 model [15], and DEMETER2 model [17]. The CERES model estimated the gene essentiality score *g_ij_* by correcting for the bias of gene copy numbers in CRISPR screens at the sgRNA level. Namely, the observed depletion score *D_kj_* for a sgRNA *k* in cell line *j* can be modelled as a linear function of the gene knock-out effect $h_{i}+g_{ij}$ and copy number effect $f_{j}\left(\sum_{l\in L_{k}}C_{lj}\right)$, as well as the sgRNA-specific error term *o_k_* and random noiseϵ:

(8) $$D_{kj}=\left(\sum_{i\in G_{k}}\left(h_{i}+g_{ij}\right)+f_{j}\left(\sum_{l\in L_{k}}C_{lj}\right)\;\right)+\;o_{k}+\epsilon$$

The DEMETER1 model estimates the gene essentiality score by adjusting for the off-target effect of shRNAs based on seed complementarity, i.e. the observed depletion score *H_kj_* for a shRNA *k* in cell line *j* can be decomposed into a sum of the gene knock-down effect *G_lj_* and seed-specific effect *S_bj_*:

(9) $$H_{kj}=\;\sum_{b\in \text{seed}\left(k\right)}\alpha_{kb}S_{bj}+\;\sum_{l\in \text{gene}\left(k\right)}\beta_{kl}G_{lj}+\;\mu_{k}+\epsilon_{kj}$$

The DEMETER2 model extends the DEMETER1 model by including the shRNA on-target efficacy *t_k_*, off-target efficacy *e_k_*, the screen signal parameter for cell line *q_j_*, and additional off-target effect *c_k_*. Furthermore, additional parameters *a_j_, θ_j_*, and *γ_j_* were used to correct the additional batch effects for the given cell line *j*:

(10) $$H_{kj}\;=\;a_{j}+\theta_{j}+\gamma_{j}\left(q_{j}t_{k}\sum_{l\in \text{gene}\left(k\right)}\beta_{kl}G_{lj}+e_{k}\sum_{b\in \text{gene}\left(k\right)}\alpha_{kb}S_{bj}+c_{k}\right)\;+\;\epsilon_{kj}$$

Note that the CERES model aimed at adjusting confounding factors in the CRISPR screen, whereas the DEMETER 1 and 2 models aimed at improving target specificity in the shRNA screens. Both methods combine the depletion scores at the shRNA or sgRNA level to infer gene essentiality scores. In contrast, the CES model intended to derive a gene essentiality score by combining the unadjusted depletion scores at the gene level, as well as the molecular features of cancer cells. Therefore, DEMETER1/2 and CERES represent screen-specific adjustment methods, whereas CES should be considered as a data integration method that utilizes gene-level data from both CRISPR and shRNA screens as well as from molecular profiling. Despite the different techniques and data sources used in these computational models, they shared the same purpose of improving gene essentiality prediction, the performance of which can be evaluated using the method below.

### 2.5 Use of ground truth gene sets for model evaluation

The CES model was compared with screen-specific methods, including CRISPR (with or without the CERES adjustment), shRNA (with or without the DEMETER adjustment), as well as the baseline methods including SA, CES^null^ and CES^perm^. For each method, a gene essentiality score can be predicted for a given gene in a cell line, based on which the ranking of the gene in this cell line can be determined. The average ranking of the gene across all 42 cell lines was considered as the overall essentiality score.

To evaluate the accuracy of overall essentiality, the ground truth of true essential genes and non-essential genes was needed. For cancer essential genes, we looked at three datasets including i) 3804 housekeeping genes curated by Eisenberg et al. [26], ii) 360 common essential genes curated by Hart et al. [20] and iii) 31 PanCancer oncogenes curated by Bailey et al. [27] (Supplementary Tables 2–4). As the overlap between these three gene sets is limited, we considered them separately when evaluating model performance (Supplementary Figure 1). For negative gene sets, we used the 927 common non-essential genes curated by Hart et al. [28] (Supplementary Table 5) along with the 75 PanCancer tumour suppressor genes curated by Bailey et al. [27] (Supplementary Table 6). We removed ambiguous genes that were included in both cancer essential genes and cancer non-essential genes.

We used the area under receiver operating curves (AUC) as the main metric to evaluate the model performance on separating 1) housekeeping genes against common non-essential genes; 2) common essential genes against common non-essential genes; and 3) pan-cancer oncogenes against pan-cancer tumour suppressor genes. The statistical significance between two ROCs was determined using the DeLong test [29]. We also evaluated the hit rates of these methods in identifying two well-known housekeeping genes, *GAPDH* and *ACTB*, which are commonly used as loading controls in western blot and qPCR experiments [30], [31], [32]. The hit rate was defined as the percentage of cell lines in which *GAPDH* and *ACTB* were identified as essential genes at various ranking cut-offs. Model predictions were also evaluated based on strictly standardized mean difference (SSMD) [33], which measures how well the true essential genes and non-essential genes are separated by each of the methods.

In addition to essential genes that showed higher overall essentiality scores, we also determined cell-specific essential genes as those ranked at the top 100 for a given cancer cell line, whereas their average rankings across all the cell lines were lower than 5000. In particular, we focused on the novel essential genes discovered by the CES method alone, which did not show cell-specific gene essentiality by either CRISPR or shRNA-based screen alone. The novel cell-specific essential genes were plotted as a bipartite network to show the interconnections of cancer dependency.

### 2.6 Survival analysis

The newly identified cancer essential genes were tested for associations with the disease-specific survival months of cancer patients. To test the effect of AGR2 in ER-positive breast cancer patients, we retrieved the breast cancer survival data from the METABRIC study [34] available from cBioPortal (http://cbioportal.org/). Of 2509 samples, we took the samples that were labelled as ER_IHC and ER_STATUS positive, and further removed samples that were labelled as ‘Died of Other Causes’, resulting in a final set of 983 samples. Microarray-based gene expression and copy number variation data for these samples were retrieved from cBioPortal. To test the effect of SRGN on the survival of AML patients. We retrieved the patient clinical data from the BeatAML study [35]. Of the 451 patients included in the study, we removed samples that were diagnostic with cancers other than ‘Leukaemia’. For patients with more than one samples, we took the earlier diagnostic samples. Furthermore, samples that were labelled with unknown or other causes of death were discarded, resulting in a final set of 297 samples. LogCPM-based gene expression data were retrieved from cBioPortal. Disease-specific survival curves were empirically estimated using the Kaplan–Meier method and Log-rank test was used to determine the significance of the difference.

## 3 Results

### 3.1 Limited genome-scale consistency between CRISPR and shRNA screens

**Fig. 2:**
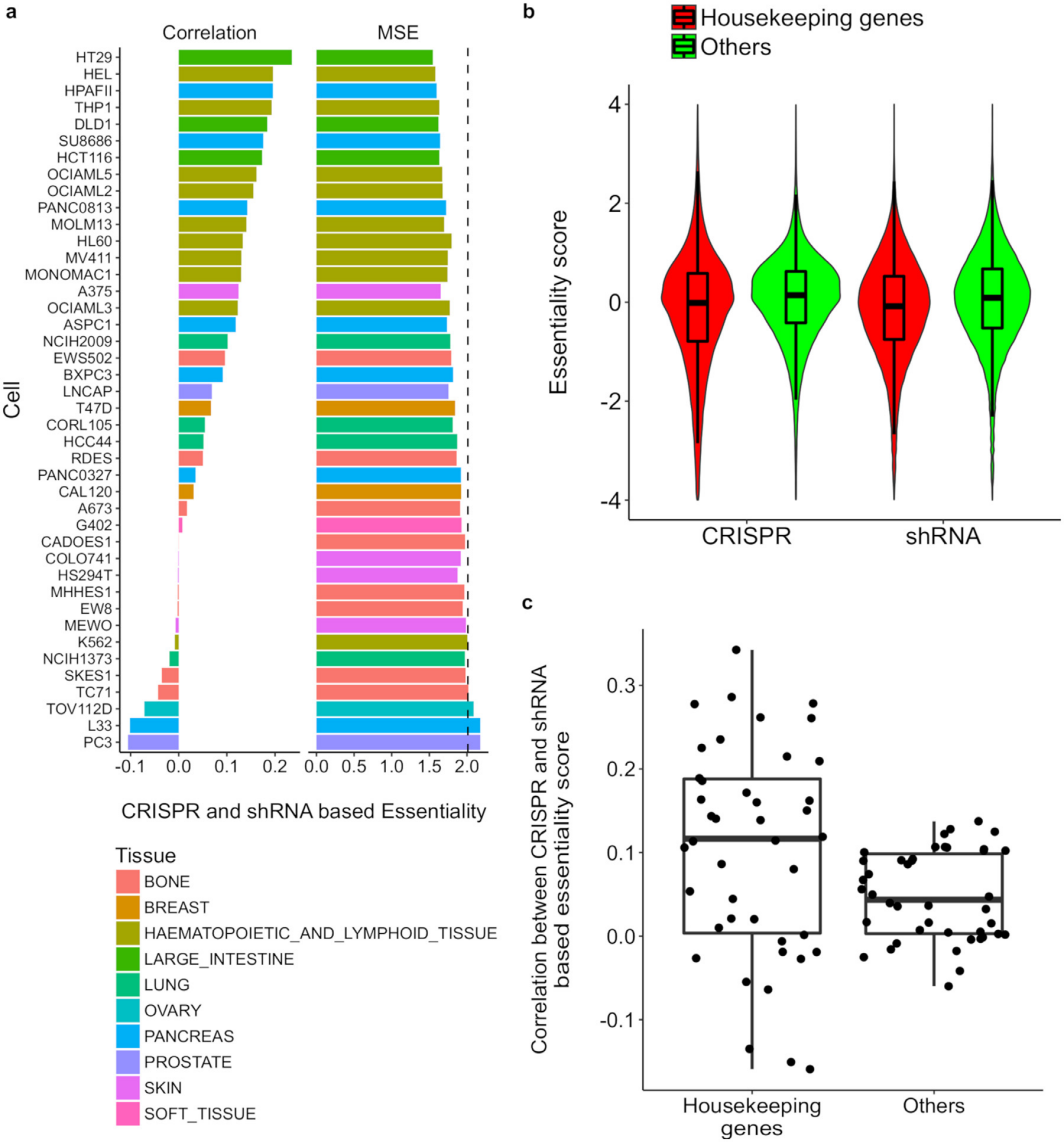
Limited consistency of CRISPR and shRNA-based gene essentiality scores across a total of 42 cancer cell lines. a. Between-screen consistency for each cell line was determined by Pearson correlation and mean squared error (MSE). The MSE for permutated CRISPR and shRNA essentiality scores is shown as the dashed line (MSE = 2.04). b. CRISPR and shRNA-based essentiality scores for the set of housekeeping genes compared to other genes. c. Pearson correlation between CRISPR and shRNA essentiality scores for the set of housekeeping genes compared to other genes.

To evaluate how the choice of technology affects gene essentiality scoring, we first evaluated the correlations of gene essentiality scores determined by CRISPR and shRNA screens for a given cancer cell line. We found generally low levels of between-screen correlations across all 42 cell lines, where 25 of them showed positive but moderate correlations (maximal correlation = 0.23), whereas 17 cell lines had negative correlations. The average between-screen correlation was 0.07, indicating a poor consistency of shRNA and CRISPR-based gene essentiality scores at the genome scale (Fig. 2a).

The HT29 cell line (colon cancer) showed the highest consistency with a between-screen correlation of 0.23. In contrast, we observed much poorer between-screen consistency for cell lines PC3 (prostate cancer) and L33 (pancreatic cancer), where the correlations dropped below zero. In general, we did not observe enrichment of certain tissue types in cell lines with higher between-screen correlations. However, 9 out of 10 leukaemia cell lines showed moderately positive correlations ranging from 0.12 to 0.20, suggesting that leukaemia cells tend to respond similarly to different screen technologies. On the other hand, the remaining leukaemia cell line (K562) had a poor correlation of −0.01, which was also replicated in a previous study [4]. We further tested the accuracy of using the shRNA score to predict the CRISPR score. The mean squared errors (MSE) were similar to those for a permutated prediction, confirming the limited genome-scale consistency of the two screens for cancer cell lines in general (Fig. 2a).

As we took the gene essentiality scores that were already summarized over multiple sequence constructs for both the shRNA and CRISPR screens, the limited consistency could not be explained simply by the biases of certain sequence constructs that may differ in their targeting efficacy and off-target tendency. On the other hand, we found that both shRNA and CRISPR screens provided lower essentiality scores (i.e. stronger gene essentiality) for housekeeping genes (*n* = 3804) compared to others (median score −0.02 vs 0.14 for CRISPR screen and −0.08 *vs*. 0.09 for the shRNA screen, respectively; Wilcoxon test p-value < 2e-16; Fig. 2b), suggesting the overall validity of the genome-wide functional screens to detect true positive hits. For these housekeeping genes, the between-screen correlations improved modestly, with an average correlation of 0.10 versus 0.05 for the other genes (Fig. 2c). However, the variation of correlations across cell lines was also inflated (variance of 0.016 versus 0.003 for the other genes). Notably, for cell lines (*n* = 13) with negative between-screen correlations, the consistency for housekeeping genes became even worse, with an average correlation of −0.026 compared to −0.018 for other genes. Similar results were also found on the common essential gene set and the PanCancer gene set (Supplementary Figure 2). These results implied that cancer essential genes did not necessarily show higher consistency between the shRNA and CRISPR screens.

### 3.2 CES improves the prediction of cancer essential genes

Given the limited consistency observed between the shRNA and CRISPR gene essentiality profiles, we developed a CES model that considers both screening technologies for estimating gene essentiality. More importantly, the CES model also included the molecular features of cancer cells to derive a more accurate gene essentiality estimation. Specifically, the CES score is a weighted average of shRNA and CRISPR gene essentiality scores, where the molecular features of cancer cells influence the weights determined by the objective function that minimizes the sum of the squares of CES and CRISPR scores (see Materials and Methods for details).

**Fig. 3:**
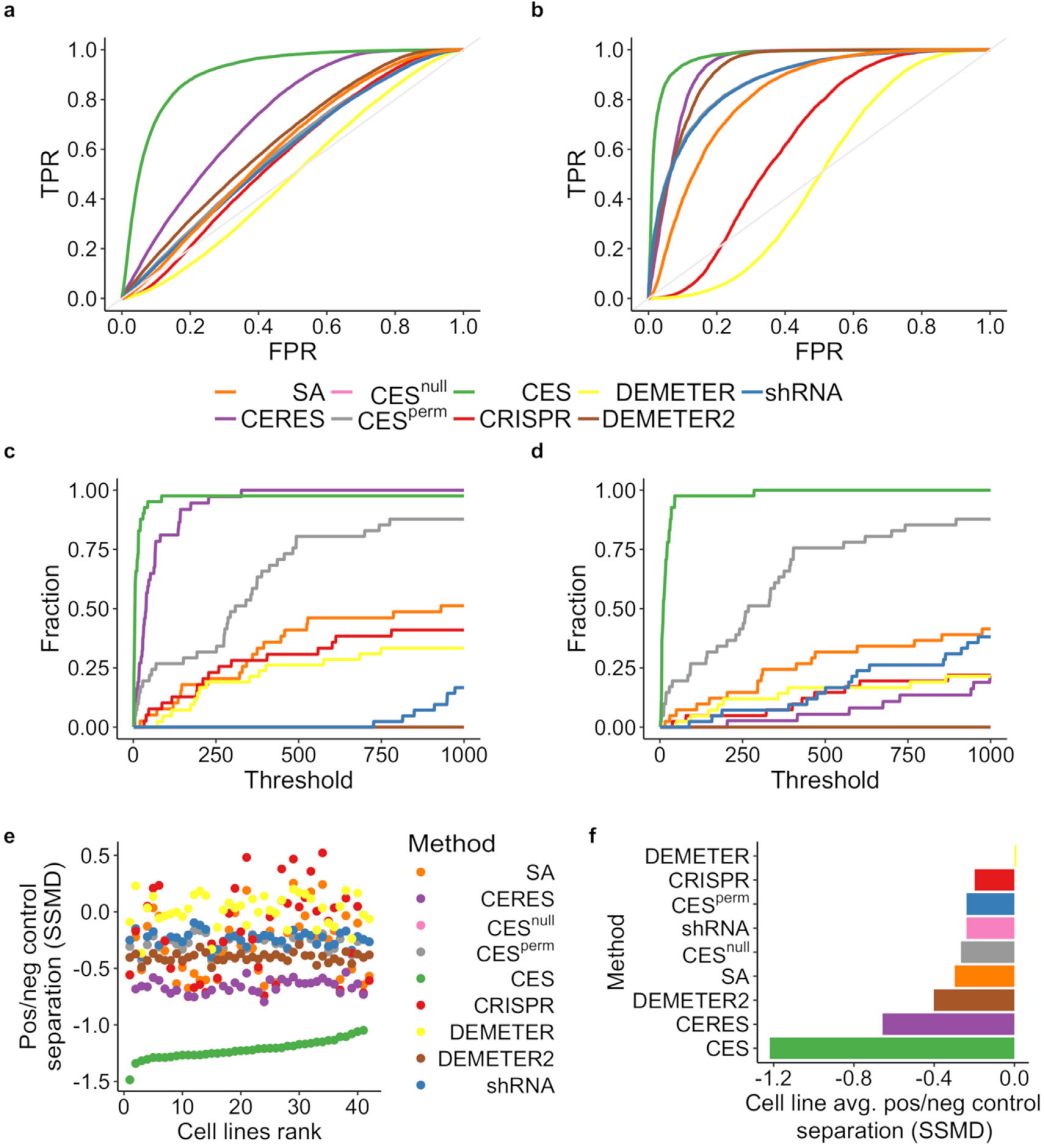
CES improves gene essentiality prediction on gold standard datasets. ROC curves were determined for each scoring method in detecting (a) housekeeping genes and (b) common essential genes. The AUC and their p-values can be found in Supplementary Table 7. The performance was illustrated using two well-known housekeeping genes including (c) *GAPDH* and (d) *ACTB*, where the fraction of cell lines in which the gene was identified as essential is shown as a function of the ranking threshold. Furthermore, the separation of gene essentiality scores for housekeeping genes and common non-essential genes was measured by the strictly standardized mean difference (SSMD), shown at (e) individual cell lines (ranked by SSMD of CES scores) and (f) averaged across all cell lines. Their p-values for median differences can be found in Supplementary Table 8.

We compared CES with baseline methods including SA, CES^null^, CES^perm^, as well as existing methods including CERES and DEMETER (see Materials and Methods for the description of these methods). The unadjusted shRNA and CRISPR essentiality scores were also included in the comparison as baseline methods. To evaluate the model performance, we used previously curated gold standards on positive cases including a housekeeping gene set (*n* = 3804), a common essential gene set (*n* = 360), and a PanCancer oncogene set (*n* = 31), as well as gold standards on negative cases including a common non-essential gene set (*n* = 927) and a PanCancer tumour suppressor gene set (*n* = 75). These gold-standard gene sets have been manually curated and widely utilized for the evaluation of functional screening results (e.g. [11], [17], [36], [37], [38]) (Supplementary Tables 2–6). We found that the CES score outperformed other scores in terms of classification accuracy for all three cancer essential gene sets (Fig. 3a-b, Supplementary Figure 3, Delong test, p-values are reported in Supplementary Table 7). For example, the area under the ROC curve (AUC) for CES on detecting housekeeping genes was 0.906, compared to 0.732 for CERES and 0.634 for DEMETER2 as the other top performing methods. As expected, the SA, CES^null^, and CES^perm^models performed relatively poorly with an AUC of 0.586, 0.596, and 0.604 separately, as these models did not include molecular features to predict gene essentiality. The performance of shRNA-based methods was generally worse than that of CRISPR-based methods. Particularly, the AUC for DEMETER is just below 0.5, suggesting that its performance is no different than a random prediction. Similarly, CES also achieved the highest accuracy to detect the common essential genes (AUC = 0.971, Fig. 3b) and PanCancer genes (AUC = 0.702, Supplementary Figure 3).

We also found that commonly known housekeeping genes were more likely to be identified by CES than by other methods. For example, *GAPDH* is a constitutively expressed gene that encodes the enzyme for regulating cell energy metabolism, the inhibition of which leads to apoptosis. In our analysis, *GAPDH* was ranked in the top 250 for 40 cell lines using CES scoring, whereas in most of the other scoring methods, the gene was essential in less than 20 cell lines even with a much looser threshold of 1000. Similar results were found for *ACTB*, which encodes a member of the highly conserved actin protein family, and is widely involved in cell motility, structure, integrity, and intercellular signalling [39]. As illustrated in Fig. 3c,d, *GAPDH* and *ACTB* were identified as cancer essential genes more often by CES than by other methods at the given ranking thresholds, resulting in the largest AUC for both genes (96.5% and 97.9% for *GAPDH* and *ACTB*, respectively).

Furthermore, we evaluated the level of separation between cancer essential and non-essential gene sets using strictly standardized mean difference (SSMD) [33]. Ideally, a scoring method that can better separate cancer essential genes from non-essential genes should result in higher absolute values of SSMD. As shown in Fig. 3e, CES scores led to a much larger separation between the housekeeping genes and the non-essential genes, outperforming other scoring methods in all the available cell lines. For example, CES increased by 85.5% on average of the absolute SSMD with robust improvement for all the cell lines compared to CERES, which was the second-best method in terms of SSMD (Fig. 3e,f, Supplementary Table 8). In contrast, methods such as raw CRISPR scores or DEMETER scores showed large variance in SSMD across different cell lines, with SSMD even being positive in some cancer cells. We also found similar results using the common essential gene set and the PanCancer gene set, although with less striking differences in SSMD (Supplementary Figure 4). Taken together, these results suggested that CES can capture the true essential genes with superior accuracy compared to the state-of-the-art methods.

### 3.3 CES corrects screen-specific biases

**Fig. 4:**
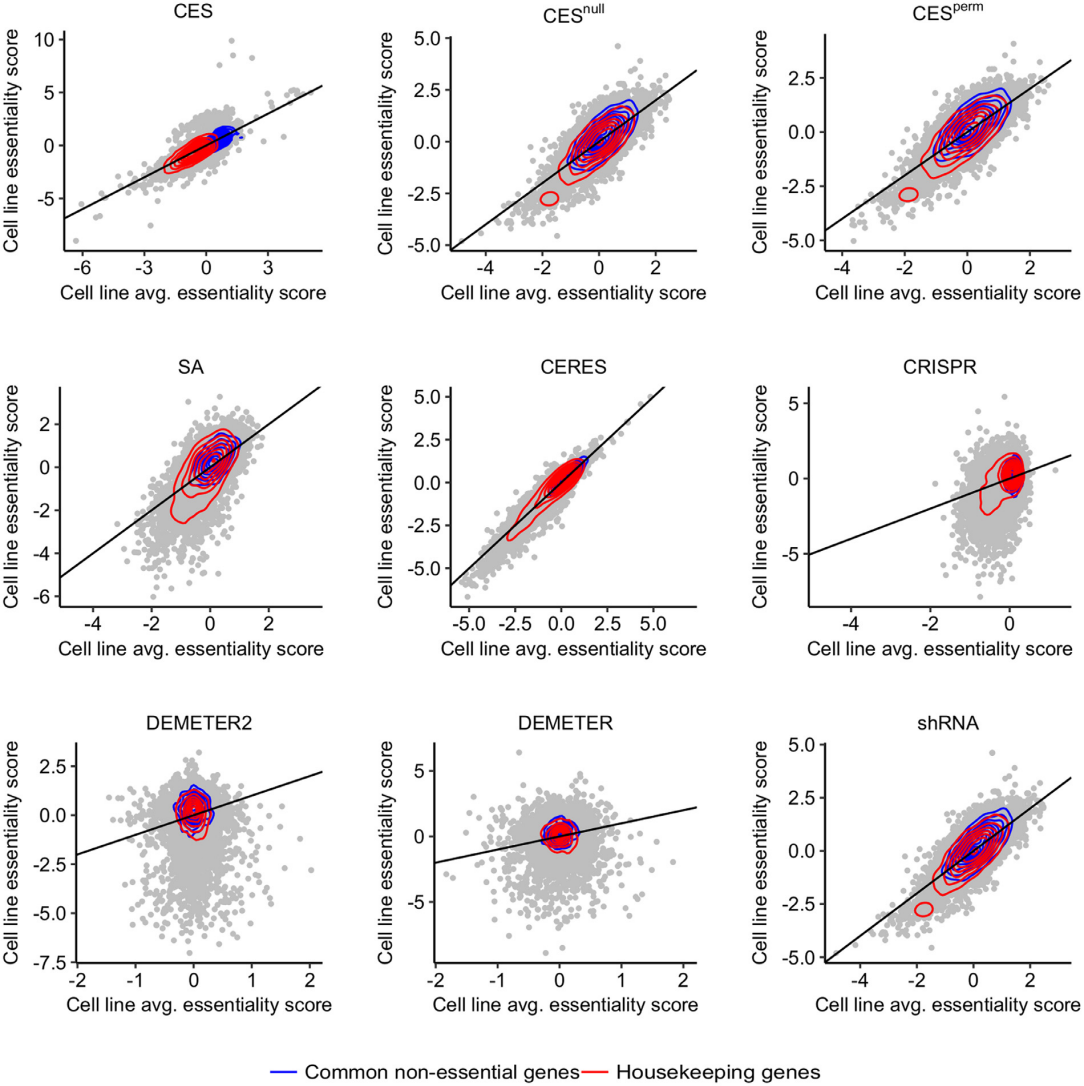
Cell-line specific gene essentiality scores versus across-cell-line average scores in HT29 cells. CES showed the clearest separation of housekeeping genes and non-essential genes compared to the other methods, highlighted by the red and blue contours as the density estimates.

As shown in Fig. 2a, the consistency of gene essentiality profiles between shRNA and CRISPR screens varied from moderate to low levels. Similar patterns were also observed for the subset of cancer essential genes (Fig. 2c). Ideally, a cancer essential gene should show higher essentiality scores across multiple cell lines, whereas a non-essential gene should be ranked at the bottom of the list. We picked up the cell line (HT29), which shows the highest between-screen consistency, and then mapped the housekeeping genes and non-essential genes on the scatter plot of the cell line average essentiality score versus the cell line specific essentiality score, determined by each of the methods (Fig. 4). CES essentiality scores separated the housekeeping and non-essential genes sufficiently well. In contrast, there was a big overlap of density estimates for the other methods, indicating that the shRNA and CRISPR screens are biased to detect certain subgroups of cancer essential genes more easily than the others. Similar patterns were also found for the common essential genes and PanCancer oncogenes versus PanCancer tumour suppressor genes (Supplementary Figure 5). These results suggest that CES scores were able to correct screen-specific biases, resulting in more centralized and separated clusters for cancer essential genes and non-essential genes. A similar pattern was observed in other cell lines, even with poor between-screen consistency such as the PC3 cell line (Supplementary Figure 6). These results suggested that the gene essentiality scores estimated by CES are more robust for the screen-specific experimental biases that are otherwise difficult to adjust using the other methods.

**Fig. 5:**
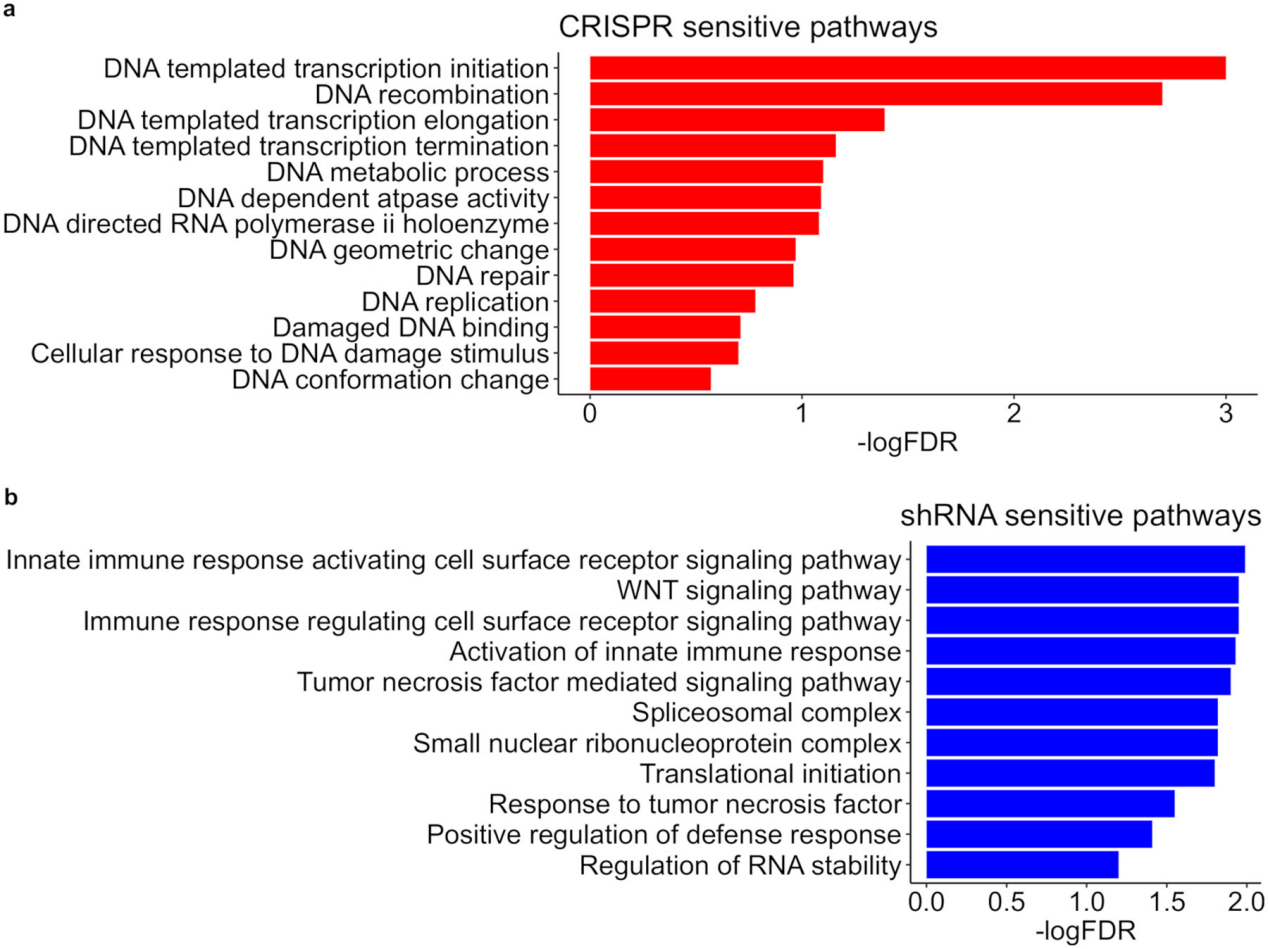
Biological pathways that are enriched in (a) CRISPR-sensitive housekeeping genes and (b) shRNA-sensitive housekeeping genes. CRISPR-sensitive housekeeping genes are defined as those that showed significantly stronger gene essentiality in CRISPR screens versus shRNA screens, whereas shRNA-sensitive housekeeping genes are defined oppositely. LogFDR is the log10 of probability that a gene set with a given enrichment score represents a false positive, determined using the GSEA method [41]. A higher –logFDR, i.e. a lower FDR indicates less false positive rate.

Recent studies have shown that cancer cells may respond to genetic perturbations introduced via shRNA or CRISPR-Cas9 by activating distinct compensation mechanisms that involve different housekeeping genes [16], [40]. As a result, housekeeping genes that showed limited between-screen consistency may be involved in these compensation mechanisms that are specific to one screen but not the other. Therefore, analysing the between-screen consistency of housekeeping genes may provide novel insights on pathways that tend to be affected by screen-specific compensation mechanisms. We focused on a subset of housekeeping genes that showed significant differences in shRNA-based essentiality scores versus CRISPR-based essentiality scores (two-group paired *t*-test, p-value < 0.05, Supplementary Table 9). We ranked these housekeeping genes (*n* = 1937) according to the difference between CRISPR scores and shRNA scores and determined the biological pathways enriched at the top or bottom ranking using the GSEA pre-ranked method [41]. The top-ranking genes in general showed strong negative shRNA scores and close-to-zero CRISPR scores, suggesting its selective sensitivity to shRNA perturbation but not CRISPR perturbation. On the contrary, the bottom ranking genes showed selective sensitivity to CRISPR perturbation. The gene set enrichment analyses showed distinct pathways that are enriched in CRISPR-sensitive housekeeping genes versus shRNA-sensitive housekeeping genes (Fig. 5). For example, CRISPR-sensitive genes are enriched mainly in DNA synthesis/metabolic (such as DNA template transcription initiation and elongation as well as DNA metabolic pathways) and DNA damage/repair related GO terms. CRISPR-Cas9 based screening perturbs cancer cells by cutting the DNA and inducing loss-of-function genetic mutations. These pathways, despite being important for cell survival, may be constitutively activated due to CRISPR-Cas9 perturbation, but not necessarily in shRNA-based screening, and may therefore explain the limited consistency of gene essentiality profiles between them. In contrast, we found significant enrichment for immune response pathways in shRNA-sensitive genes, including WNT and tumour necrosis factor signalling pathways. These pathways tend to respond to shRNA perturbations but not CRISPR perturbations. Interestingly, siRNA-associated immune stimulation has been described previously [42]. Taken together, these results suggest that the shRNA and CRISPR screens may activate specific biological processes that are independent of the true essentiality of the intended target genes. These distinctive biological processes that are activated in one form of genetic perturbation but not the other, may be worth further exploration with in-depth biological validation (Supplementary Tables 10,11).

### 3.4 CES identifies the molecular biomarkers for cancer essential genes

For a given cancer essential gene, we also applied a linear regression model to explain the CES using its molecular features including genetic mutation, gene expression, and copy number. A significant molecular feature may therefore be considered as a biomarker for gene essentiality, for which the coefficient can be interpreted as the weight of the biomarker. Note that a lower CES indicates a stronger gene essentiality by convention, a negative weight therefore suggests a positive influence of the molecular feature contributing to the gene essentiality.

**Fig. 6:**
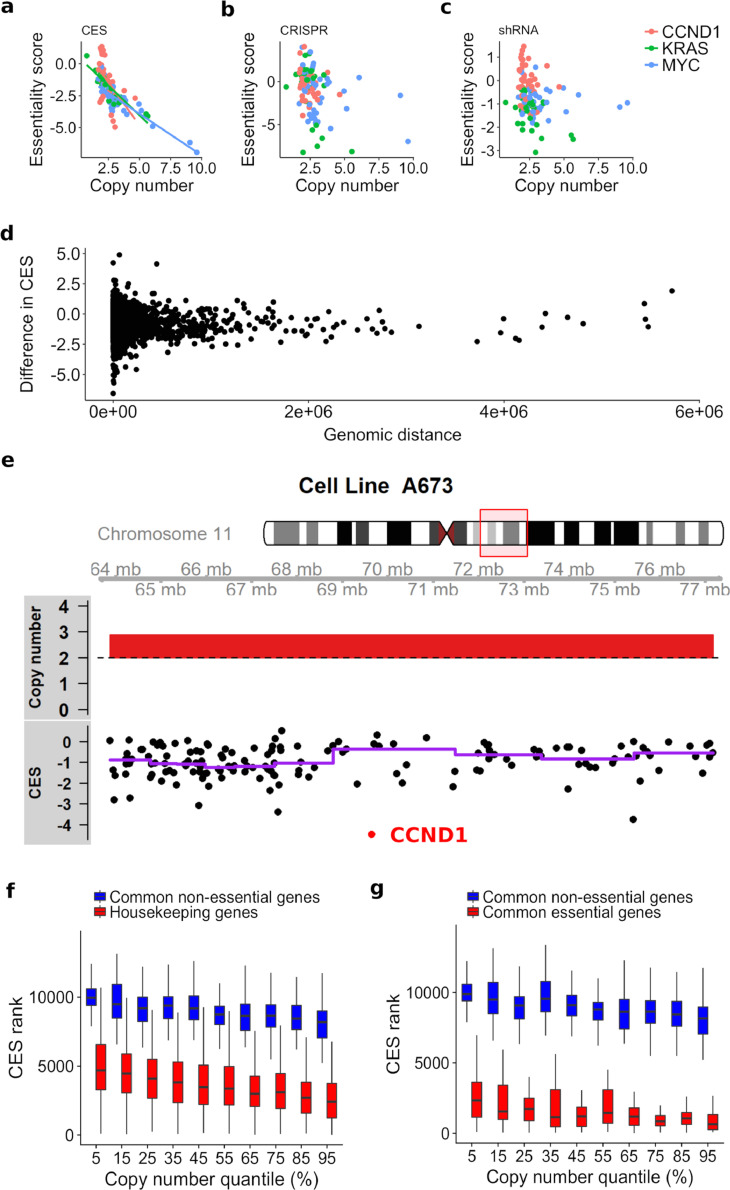
The predicted CES essentiality in genomic amplified regions. (a–c) Increased essentiality driven by a higher copy number in oncogenes *KRAS, MYC*, and *CCND1*, captured by CES but not by CRISPR or shRNA scores. (d) Difference of CES for cancer essential genes and their neighbouring genes does not correlate with their genomic distances. (e) *CCND1* was predicted as a top essential gene by CES from the amplified 11q13 genomic region in cell line A673. (f,g) CES separates housekeeping genes (f) and common essential genes (g) from non-essential genes under the same scenario of copy number quantiles.

We found that most of the cancer essential genes (i.e. housekeeping genes, common essential genes and PanCancer oncogenes) showed significant weights (with nominal p-value < 0.05) for copy number variation, accounting for 46.0% of all the significant weights, followed by microarray-based gene expression (31.9.%), RNA-Seq-based RPKM gene expression (17.7%), and mutation (4.4%). The weights for the copy number feature are negative (−0.53 on average), suggesting that for a given gene, the possibility of the gene being essential increases with an increasing copy number. Copy number alterations are known to be the most frequent genetic changes in cancer cells [43]. We reasoned that gene copy amplification increases the chromosomal instability correlated with the disease state and prognosis, resulting in the activation of genes with enhanced essentiality [44], [45], [46]. For example, oncogenes including *MYC, KRAS,* and *CCND1* showed strong CES with increasing copy numbers, corroborating the recent studies about cancer dependency on these gene amplifications (Fig. 6a) [47]. In contrast, neither CRISPR nor shRNA-based methods could capture such a pattern clearly (Fig. 6b,c). The weights of gene expression features are also generally negative using microarray (−0.62 on average) and RPKM (−0.46 on average), suggesting that if a gene is upregulated, it is more likely to be essential, which was also reported in recent large-scale RNAi studies [17].

CRISPR screens tend to produce false positive estimates of gene essentiality for non-essential genes that are amplified in the same region with cancer essential genes [11]. Therefore, we also evaluated whether CES tends to be affected by the same bias. We investigated CES for cancer essential genes and their neighbouring genes located in the same genomic regions. Specifically, for a given essential gene-neighbour gene pair, we measured the distance in base pairs as well as the difference between their CES. We found zero correlation between the genetic distances and CES difference, suggesting that the CES is unlikely to predict a higher gene essentiality simply due to copy number amplification (Fig. 6d). For example, *CCND1* is located in cytogenetic band 11q13, and is predicted as the top essential gene among its neighbours located in the same amplified region (Fig. 6e). Similarly, *KRAS* and *MYC* are also ranked at the top within highly amplified genomic regions (Supplementary Figure 7). At the genome-level, we found that CES could separate cancer essential genes from common non-essential genes across all copy number levels (Fig. 6f,g and Supplementary Figure 8). These lines of evidence suggested that CES is not biased by copy number amplification, which is known to be a confounding factor in CRISPR-Cas9 screens [9], [12], [48].

Despite the relatively low number of significant coefficients for mutation features, CES recapitulated the well-known mutation dependency for multiple oncogenes. We ranked the genes by the significance of their mutation weights and found the top three genes being key components of the RAS family including *NRAS* (estimate = −0.14, p-value = 5.61e-10), *KRAS* (estimate = −0.09, p-value = 1.47e-7) and *BRAF* (estimate = −0.06, p-value = 1.18e-5). For example, CES ranked *NRAS* at the top in two AML cell lines including THP1 and HL60, where *NRAS* is highly mutated with 15- and 21-fold greater frequency than the average. This suggested that *NRAS* mutation might play a crucial role in the proliferation and survival of AML. Indeed, oncogenic *NRAS* mutations are highly prevalent in AML patients [49] and MEK inhibitors targeting oncogenic N-Ras signalling are currently under clinical trials for AML patients [50]. Taken together, the regression of CES on molecular features may provide functional links between the genotype-phenotype of cancer essential genes that may be worth exploring as biomarkers and drug targets.

### 3.5 CES identifies cell-specific gene essentiality

**Fig. 7:**
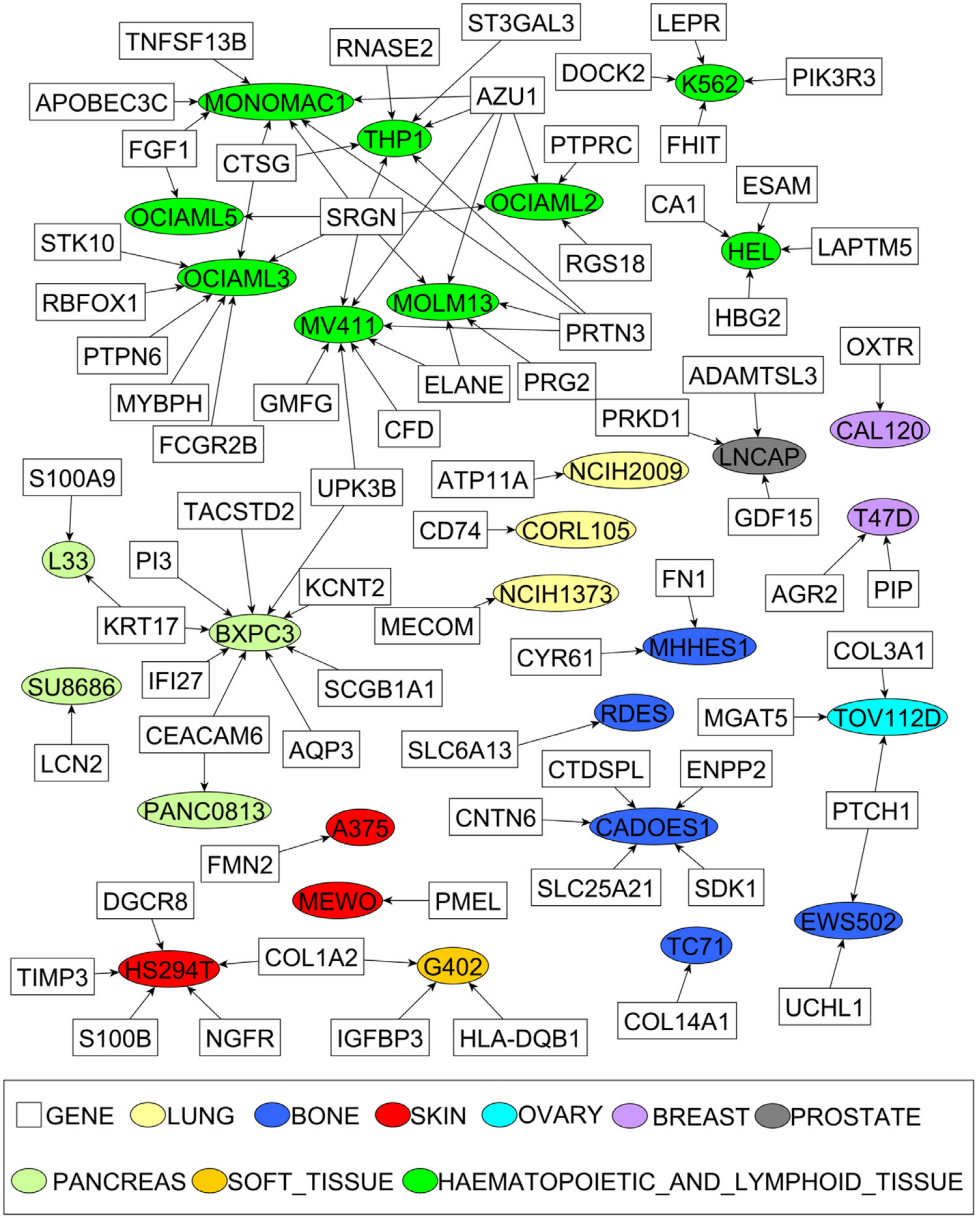
The novel cell-specific essential genes detected exclusively by CES. Round nodes are cell lines and square nodes are genes.

Finally, we utilized the CES to identify cell-specific essential genes, defined as genes ranked in the top 100 for a given cancer cell line, while their overall rankings across all cell lines went below 5000. Altogether 219 genes for the 41 cell lines, including 287 cell-gene pairs were detected (Supplementary Figure 9). In particular, we were interested in novel essential genes discovered by the CES method but not by the CRISPR and the shRNA-based methods. Therefore, we focused on the subset of cell-specific genes that ranked below 2000 in both CRISPR and shRNA-based scoring. Altogether, 68 such novel essential genes were identified for 29 cell lines, for which the cancer dependency network is shown in Fig. 7. We reasoned that cell-specific essential genes should generally have higher expressions in a cell-specific manner as well. Indeed, the expression of essential genes in these cell lines is significantly higher than that of other cell lines (average rank 6.07 *vs*. 24.70, *p*-value < 2.2e-16 for RNA-seq based expression; average rank 5.46 *vs*. 25.66, p-value < 2.2e-16 for microarray based expression, Wilcoxon rank sum test).

**Fig. 8:**
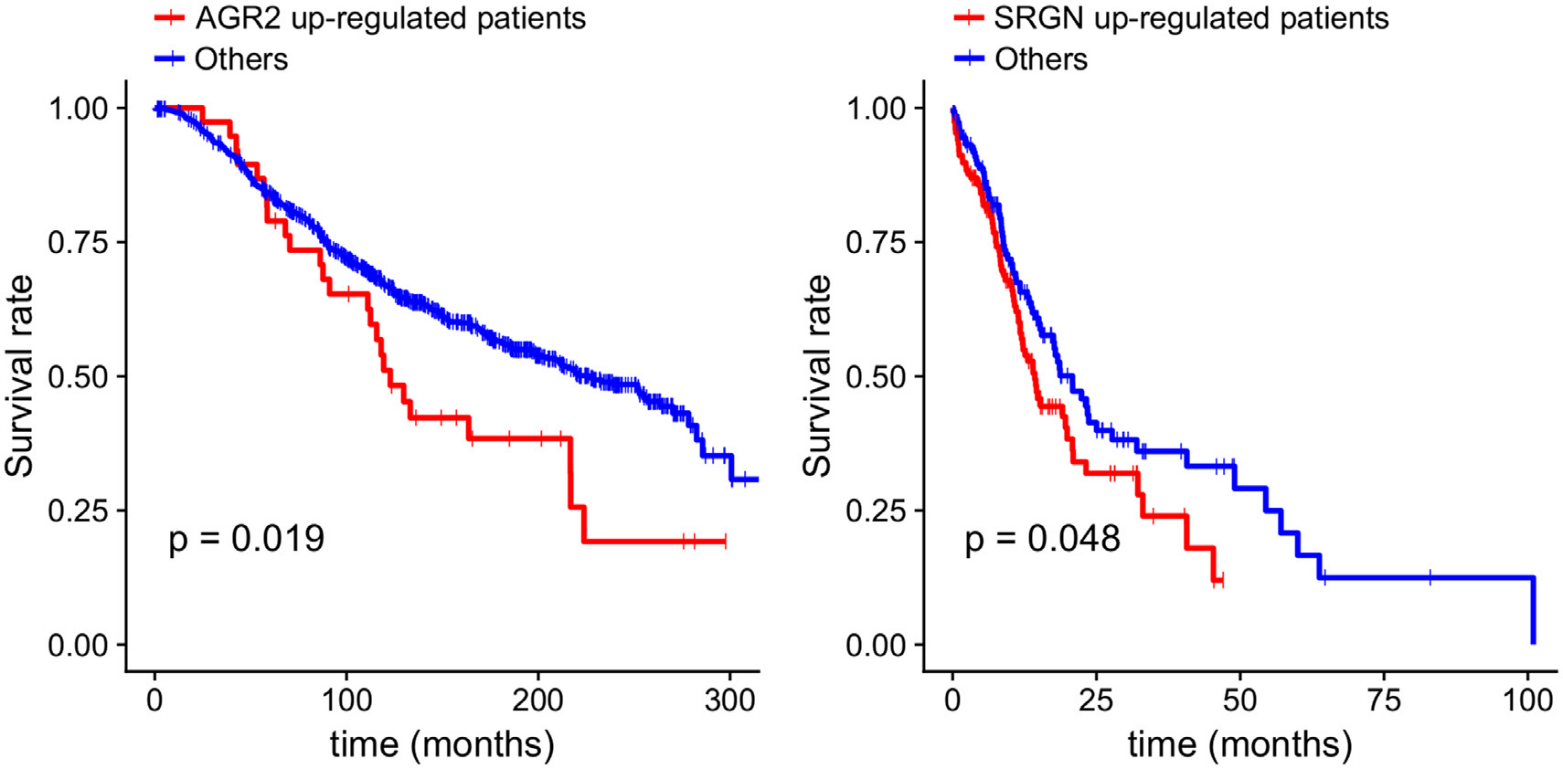
Survival analysis for the novel cancer essential genes including a) AGR2 in ER-positive breast cancer patients and b) SRGN in AML patients. Disease-specific survival curves were empirically estimated using the Kaplan–Meier method and Log-rank test was used to determine the significance of the difference.

The majority of the genes were essential in only one cell line by the cell-specific selection criteria. For example, *AGR2* has been identified by CES as an essential gene for the T47D cell line (breast cancer) but not others. *AGR2* has been reported to play a critical role in oestrogen receptor (ER) positive breast cancer development [51]. In contrast, both the CRISPR and shRNA screens failed to identify *AGR2* as an essential gene for the T47D cell line (essentiality score = 0.24 and 0.35, respectively). However, certain essential genes were shared by multiple cell lines, suggesting an extended level of essential gene specificity for a group of cancer cell lines, which might allow them to be used as predictive biomarkers for patient stratification. For example, *SRGN* that encodes a hematopoietic cell granule proteoglycan, has been identified by CES as a cell-specific essential gene for eight leukaemia cell lines, of which seven were not detected by CRISPR or shRNA-screens alone, including MOLM13, MV411, MONOMAC1, OCIAML2, OCIAML3, OCIAML5, and THP1. Interestingly, all these cell lines belong to the AML (acute myeloid leukaemia) subtype, suggesting the potential of using *SRGN* as a prognostic biomarker or drug target for AML. Indeed, as shown in Supplementary Figure 10, *SRGN* expression was higher in these AML cell lines. Using patient data from the METABRIC and BeatAML studies (see Methods for details), we further showed that *AGR2* upregulation is predictive of poor survival in ER-positive breast cancer patients, and that *SRGN* upregulation is associated with AML prognosis (Fig. 8). More specifically, for the 983 ER-positive samples in the METABRIC study, we found that samples (*n* = 38) with higher gene expression (EXP z-score >2) or copy number amplification (CNV > 1) showed a significantly poor prognosis (median disease-specific survival 123 months vs. 226 months, log-rank test, *p* = 0.02) compared to other samples; For the 297 AML samples in the BeatAML study, we found that patients with high *SRGN* expression (logCPM median z-score > 0, *n* = 150) had significantly poor survival compared to others (median disease-specific survival 14.3 months vs. 20.8 months, log-rank test, *p* = 0.05). These results suggested the clinical potential and benefit of targeting these essential genes for specific patient groups. The actual functions of *AGR2* and *SRGN*, together with other novel essential genes that were found specifically for specific groups of cell lines, might be worth further investigation to facilitate patient stratification and drug discovery in personalized medicine.

## 4 Discussion

Loss-of-function genetic screens with shRNA- and CRISPR-based techniques have been commonly utilized for studying cancer dependency at the genome-level, although questions remain on how to efficiently leverage these datasets to generate more consistent gene essentiality profiles for a given cancer sample. A recent side-by-side comparison in the K562 cell line (leukaemia) demonstrated a lack of consistency for the essential genes identified by these two screen techniques [4]. In this study, we performed a more systematic comparison using a panel of 42 cancer cell lines representing ten tissue types and confirmed the limited between-screen consistency across various cellular contexts. Reasons for this limited consistency might vary and may depend on confounding factors related to experimental design, as well as inherent biases that are specific to one screen technology but not the other. For example, recent studies showed that CRISPR screens may erroneously identify genes in copy-number-amplified regions as essential, as the DNA damage response and cell cycle arrest may be triggered by the CRISPR-Cas9 process independently of the essentiality of the targeting genes [2]. It has also been shown that shRNA screens may be less likely to detect essential genes expressed at low levels [20]. In our data analysis, we also found that housekeeping genes that are involved in certain biological pathways tend to respond differently to CRISPR and shRNA perturbation (Fig. 5). These and other confounding factors may contribute to the limited between-screen consistency, which may pose a challenge to estimate true gene essentiality.

To improve the accuracy of gene essentiality estimation, many computational methods have been developed for genetic screens using single technologies, whereas there is a lack of integrative methods to combine the gene essentiality profiles from both CRISPR and shRNA screens. Furthermore, the molecular features of cancer cells that are known to play important roles in determining the function of genes have not been effectively considered when estimating gene essentiality. Following this line, we proposed a data integration model called Combined Essentiality Score (CES) to integrate the genetic essentiality profiles from these two screening techniques, while accounting for the molecular signatures of cancer cells. We showed that the CES model improved the prediction of multiple reference sets of cancer essential genes and non-essential genes, compared to existing computational methods including CERES and DEMETER1/2. Furthermore, CES was able to correct the screen-specific biases, suggesting that the CES could be used as a more reliable metric for estimating true essentiality. The CES method differs from existing computational methods that usually consider one type of genetic screen. Rather, CES tries to integrate the gene essentiality profiles from both CRISPR and shRNA screens and improves gene essentiality estimation by incorporating molecular features including mutation, gene expression, and copy number variation. In contrast, CERES and DEMETER1/2 focused mainly on off-target correction, but rarely considered the differential responses to CRISPR and shRNA perturbations, which could explain the less accurate prediction performance compared to CES. Furthermore, the linear structure of CES allows the quantification of molecular feature effects on gene essentiality and may thus may provide clues about why a given gene is essential. We demonstrate several case studies where the identified cancer essential genes indeed make biological sense that may warrant further experimental validation. Importantly, we assumed that interactions between molecular features, the raw CRISPR and shRNA scores, and true essentiality scores are rather complex and most likely gene- and cancer-specific. Therefore, we reported the source code and CES scores to allow cancer researchers to further test the validity and biological rationale of CES at the genome and pan-cancer level.

We collected a total of 42 cell lines from recent publications to demonstrate our model. It should be noted that several large-scale projects including Achilles [12] and DepMap [15] are continuously generating genome-wide functional genetic screen data for more cancer cell lines. The accumulating genome-wide functional screening data are expected to improve the scope of the CES model. On the other hand, the CES model currently takes gene level essentiality scores as inputs, because gene-level data are more commonly available from existing studies. Data pre-processing procedures from the shRNA or sgRNA level to gene level may affect the CRISPR and shRNA-based gene essentiality estimation and may thus affect the CES. Given that high quality shRNA level or sgRNA level data have been made available recently, direct modelling of gene essentiality profiles from sgRNA and shRNA level data may be worth exploring as a future step.

Our method provides a novel perspective to explore the large feature space for cancer genes, allowing improved prediction of essential genes and their functional annotation in individual cell lines. Effective integration of functional and molecular data might provide important clues for drug discovery in personalized medicine. For example, we utilized the mutation status of genes as a predictor of CES score. The correlation of CES score with mutation status might be indicative of whether such a mutation is an activating or inactivating mutation. However, more experimental validation is required for evaluating its potential as a drug target. Although we have focused on high-throughput functional genetic screens that are largely measuring cell growth in this study, the CES modelling strategy itself is applicable to interrogate genes that are essential for other cellular phenotypes and functions. For example, recent technologies in single-cell sequencing have enabled the testing of multiple phenotypes for specific gene depletions in a pooled fashion (e.g. CROP-seq and Perturb-seq) and we expect that our method can be also applied there [52]. In addition to human cancer cell lines that were studied most extensively, limited phenotypic consistencies were also observed in other model organisms including mouse and zebrafish [53], [54]. We foresee that the CES model can be extended to improve the estimation of genetic dependency in cancer cell lines as well as in other model organisms.

## Declaration of Competing Interest

None declared.

## Appendix Supplementary materials

Image, application 1

Image, application 2
