# Corrigendum to ‘Combined gene essentiality scoring improves the prediction of cancer dependency maps’ [EBioMedicine 50 (2019) 66–79]

**DOI:** 10.1016/j.ebiom.2019.12.003

**Published:** 2020-01-03

**Authors:** Wenyu Wang, Alina Malyutina, Alberto Pessia, Jani Saarela, Caroline A. Heckman, Jing Tang

**Affiliations:** a Research Program in Systems Oncology, Faculty of Medicine, University of Helsinki, Haartmaninkatu 8, FI-0 0 014 Helsinki, Finland; b Institute for Molecular Medicine Finland (FIMM), University of Helsinki, Tukholmankatu 8, FI-0 0 014 Helsinki, Finland

The authors regret that there is an error in their code related to the DEMETER2 plots in Figs. 4, S5 and S6. By mistake they used the DEMETER score to calculate the cell line averaged score for DEMETER2. This error has been corrected in the Github (https://github.com/Wenyu1024/CES). This error does not change the scientific conclusions of the article.

**Fig. 4.**
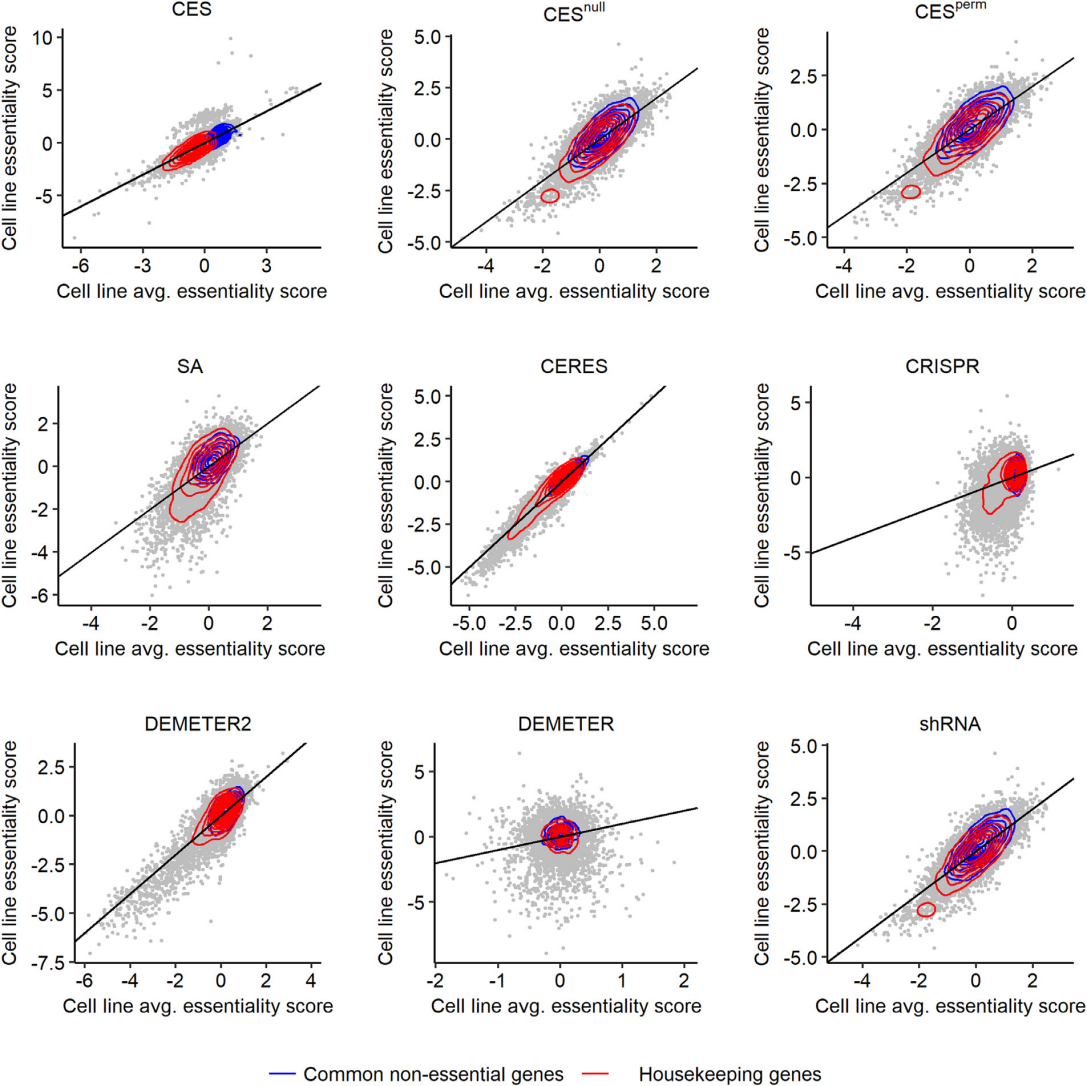
Cell-line specific gene essentiality scores versus across-cell-line average scores in HT29 cells. CES showed the clearest separation of housekeeping genes and nonessential genes compared to the other methods, highlighted by the red and blue contours as the density estimates.

The corrected plots for DEMETER2 are updated in the figures below (other subplots in Figs. 4, S5 and S6 remain unchanged).

The authors would like to apologize for this error.

## Appendix Supplementary materials

Image, application 1

