# Supplementary material for: Combined gene essentiality scoring improves the prediction of cancer dependency maps
Source: eBioMedicine. 2019 Nov 12;50:67–80. doi: 10.1016/j.ebiom.2019.10.051 (PMC6923492; doi:10.1016/j.ebiom.2019.10.051)
Supplement: Supplementary file 1 [file mmc1.docx]

**Supplementary Figures**


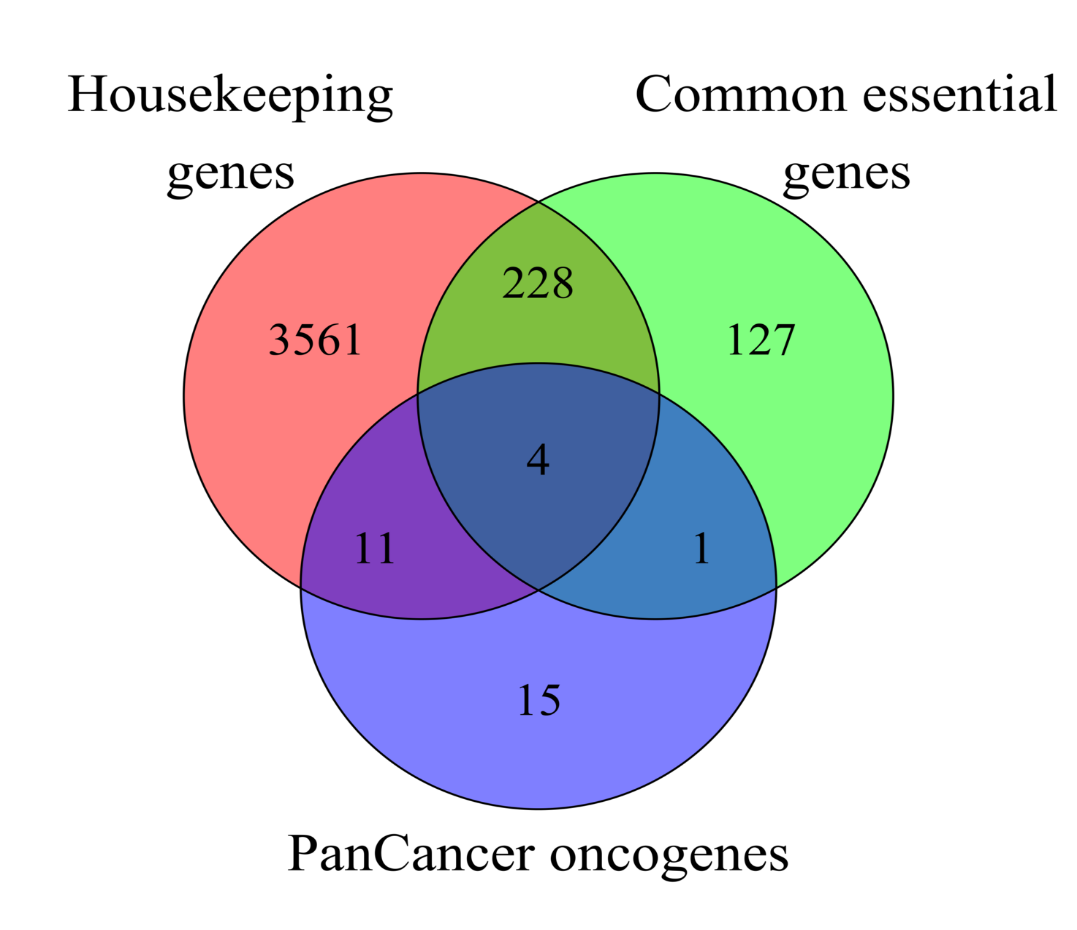


**Figure S1**. Overlap of three reference cancer essential gene sets.


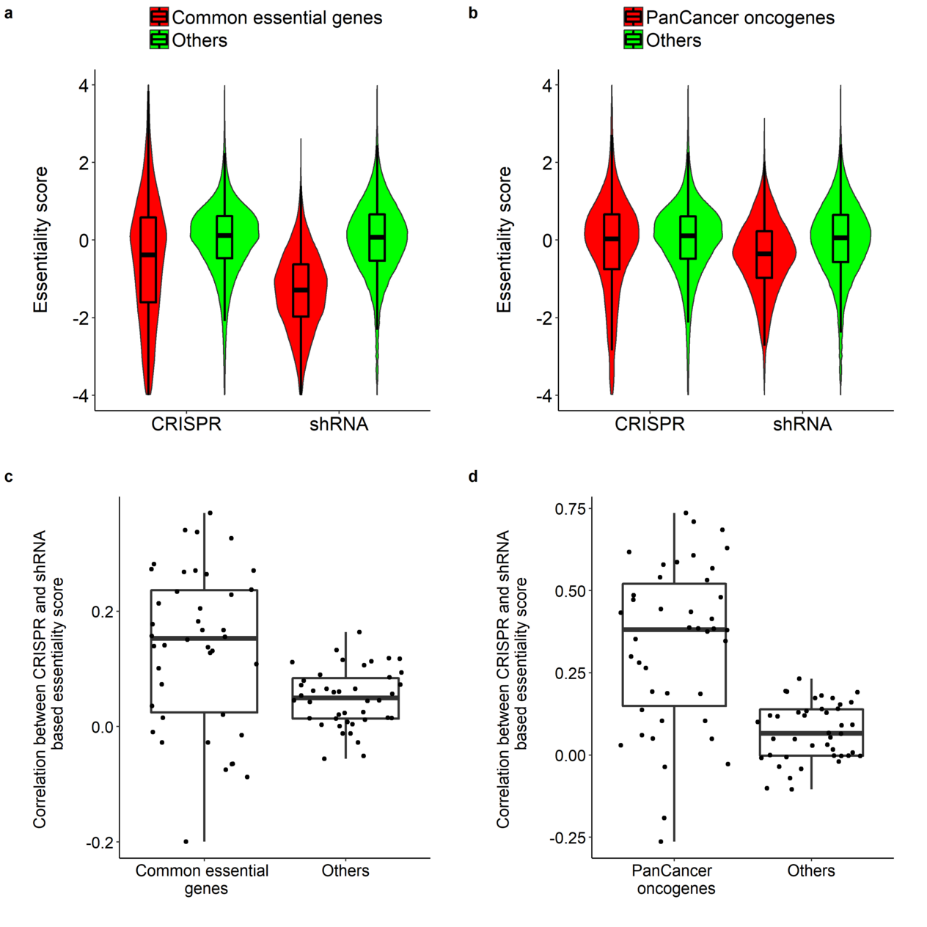


**Figure S2.** Limited consistency of CRISPR and shRNA-based gene essentiality scores in common essential genes (**a**, **c**) and PanCancer oncogenes (**b**, **d**).


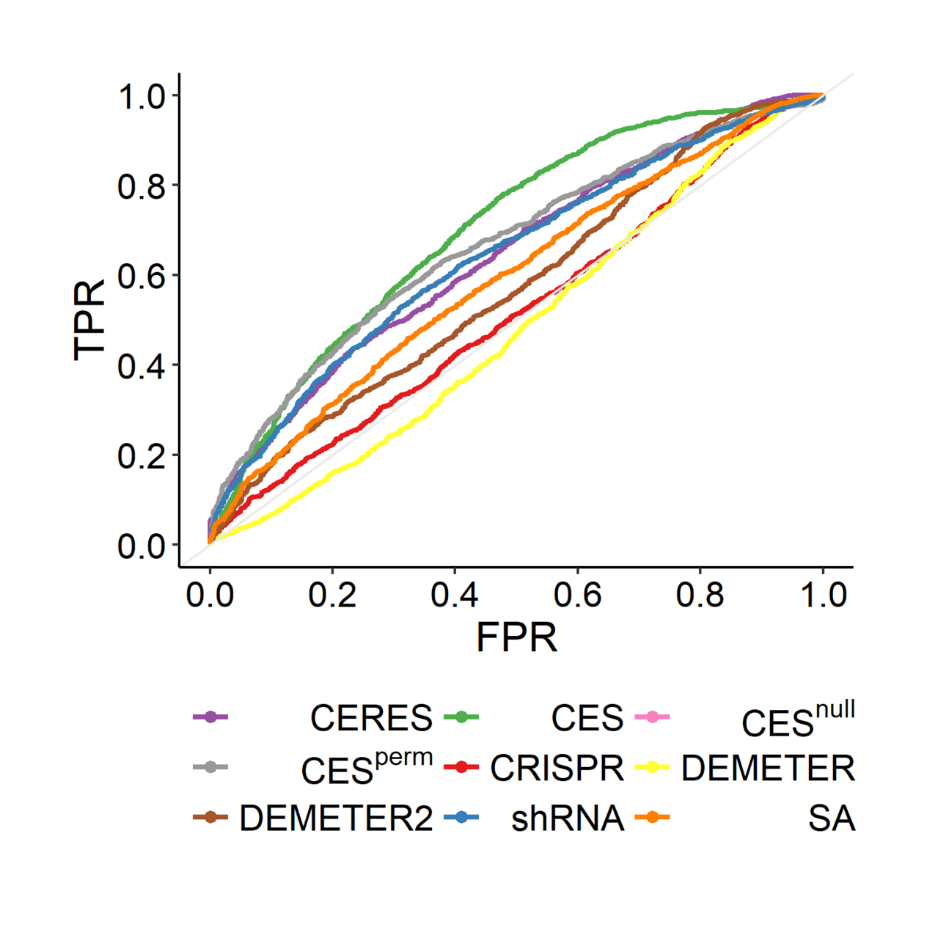


**Figure S3.** The ROC curves for each scoring method in separating the PanCancer oncogenes from PanCancer tumour suppressor genes. The AUC and their p-values can be found in **Supplementary Table S7**.


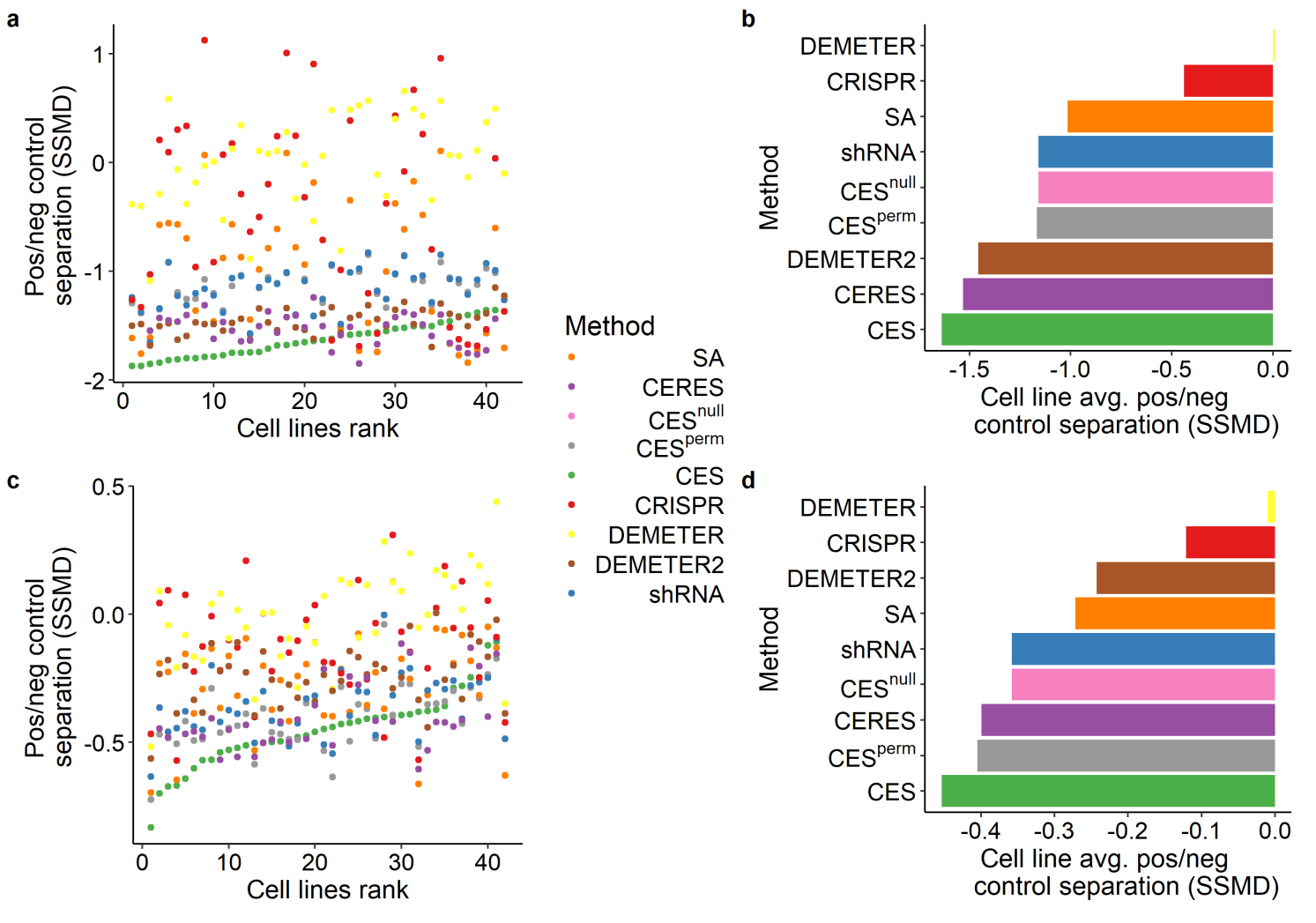


**Figure S4.** SSMD for common essential genes against common non-essential genes **(a-b)** and PanCancer oncogenes against PanCancer tumour suppressor genes **(c-d)**. Their p-values for median differences can be found in **Supplementary Table 8**.

**
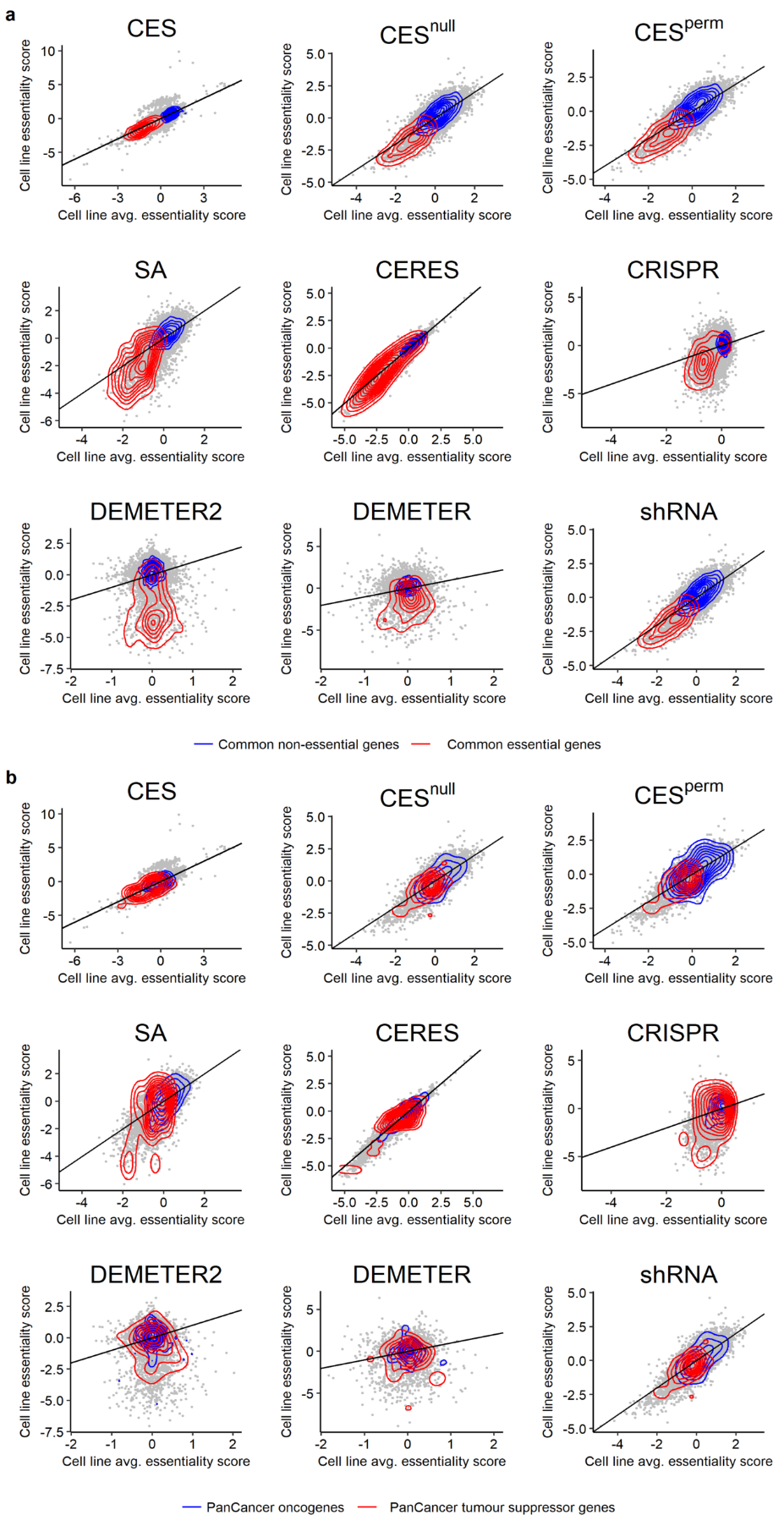
**

**Figure S5.** The density estimation of **(a)** common essential genes *vs*. non-essential genes and **(b)** PanCancer oncogenes *vs*. PanCancer tumour suppressor genes in HT29 cells. CES showed the clearest separation of these two groups compared to other methods.


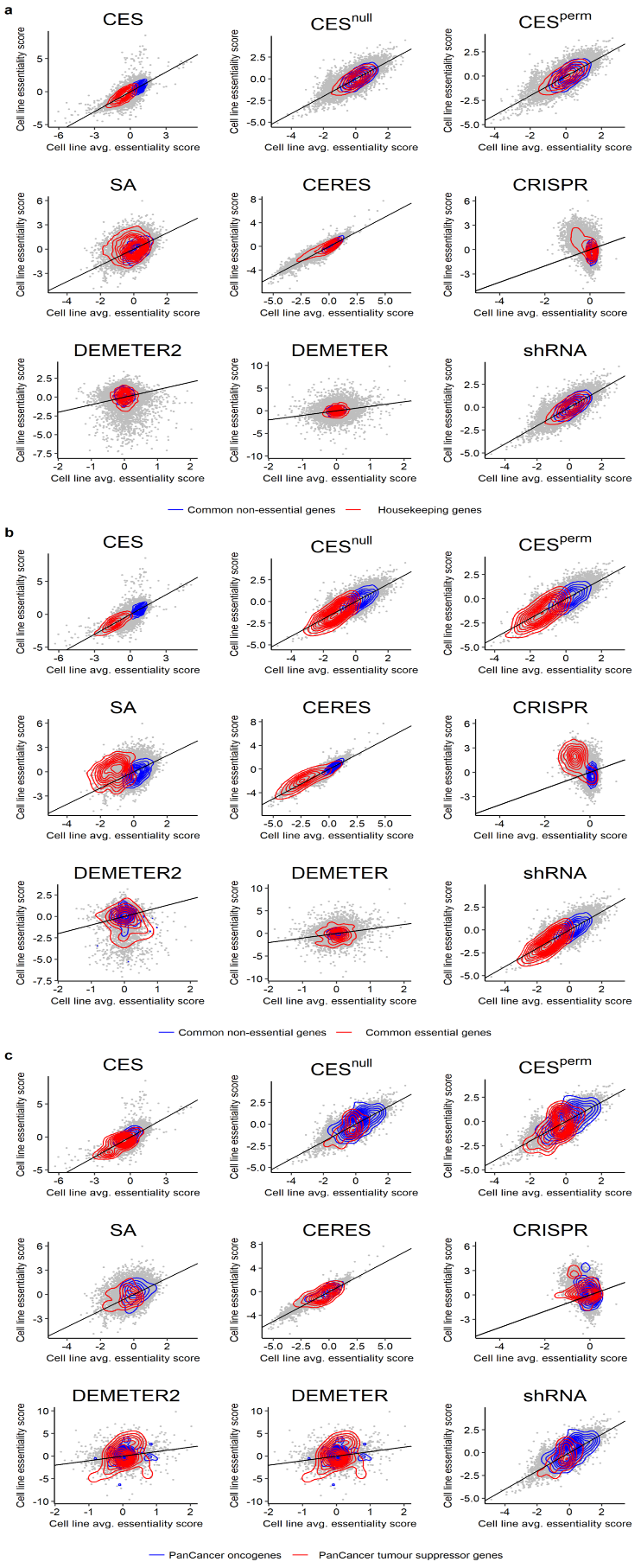


**Figure S6.** Density estimation of **(a)** housekeeping genes, **(b)** common essential genes versus non-essential genes and **(c)** PanCancer oncogenes *vs*. PanCancer tumour suppressor genes in PC3 cells. CES showed the clearest separation of these two groups compared to other methods.


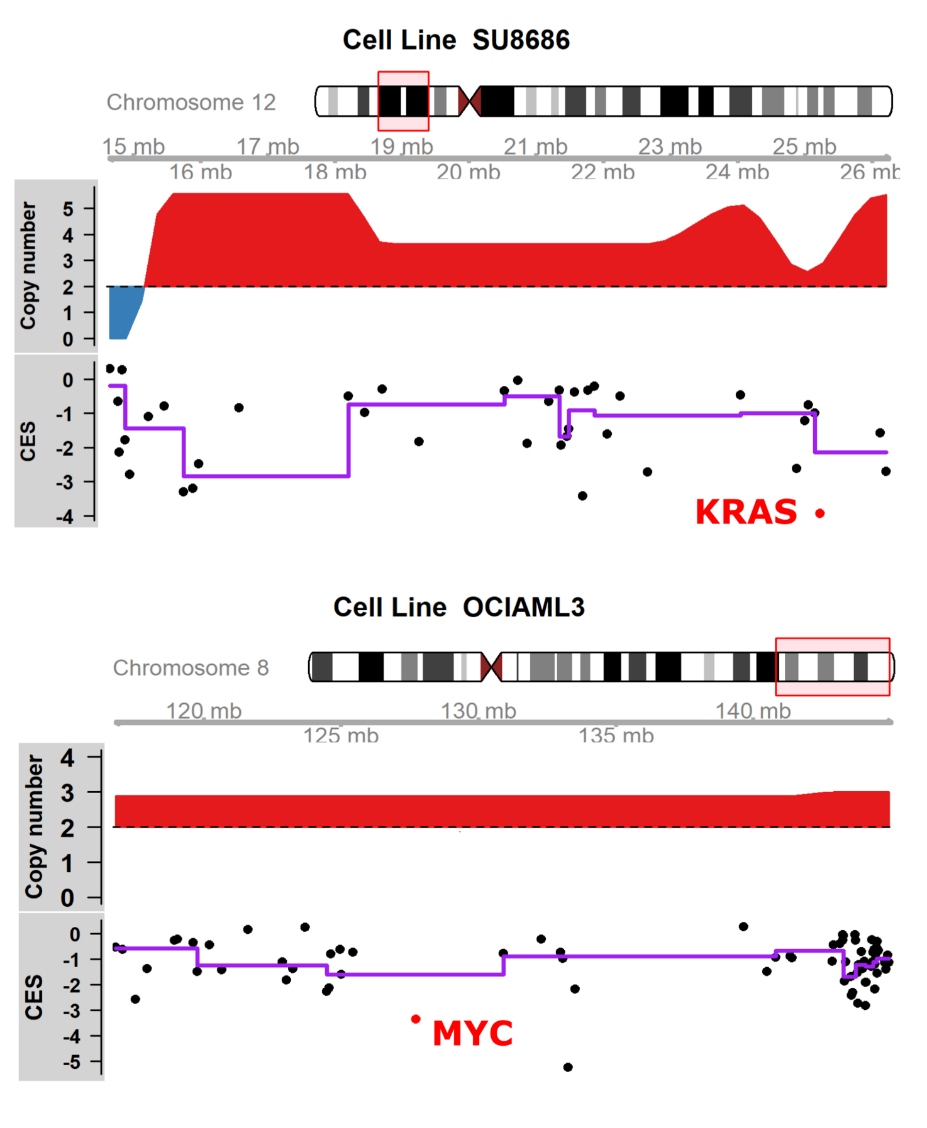


**Figure S7.** Oncogenes **(a)** *KRAS* and **(b)** *MYC* were ranked at the top in the amplified regions in SU8686 cells and OCIAML3 cells.

**
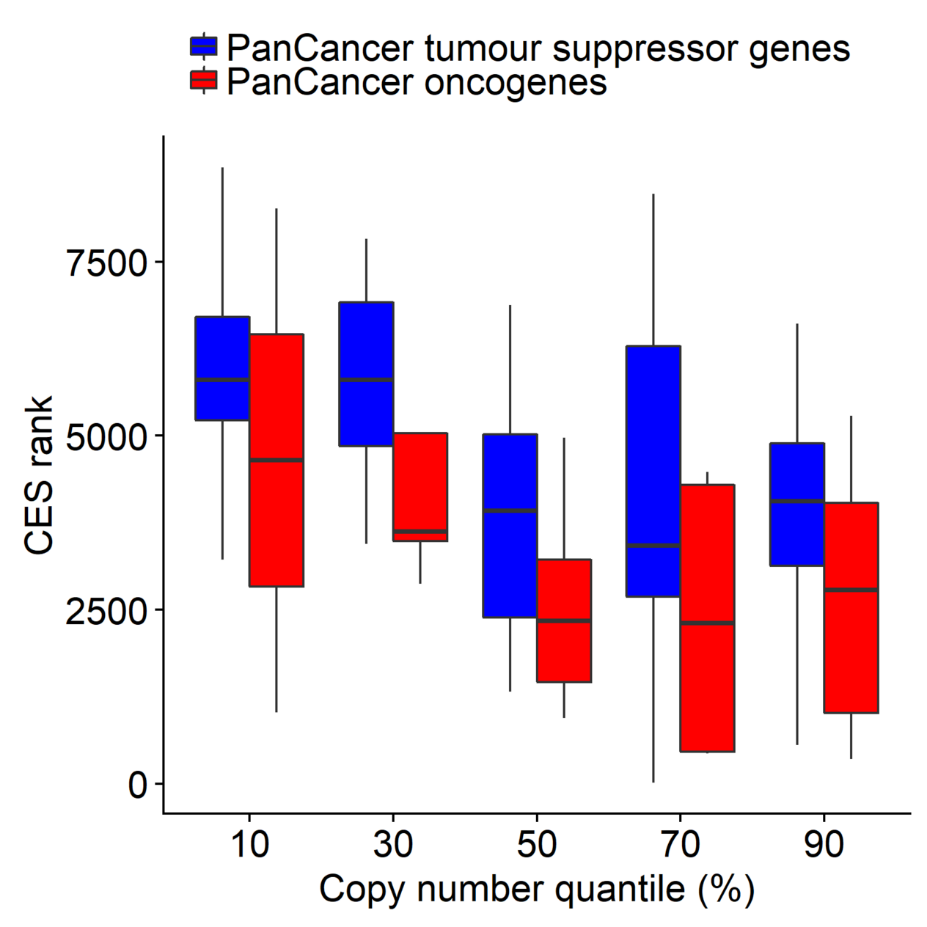
**

**Figures S8.** CES separates PanCancer oncogenes from PanCancer tumour suppressor genes under different scenarios of copy number quantiles.


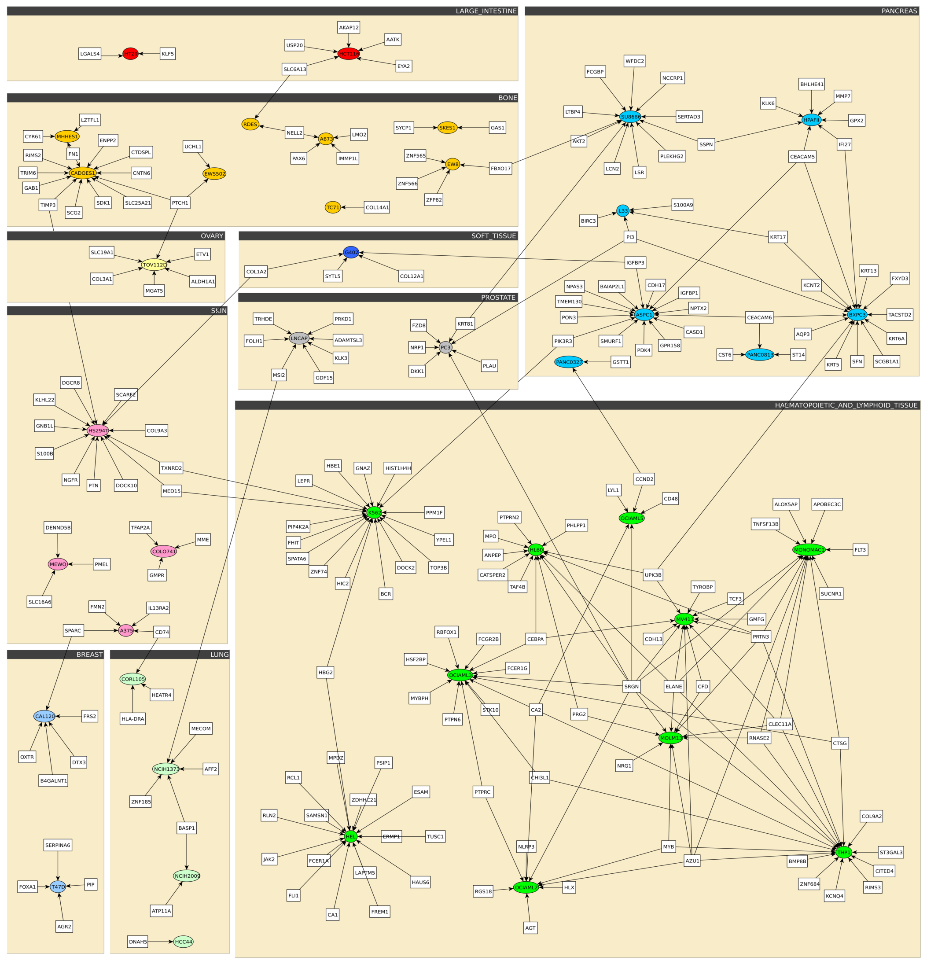


**Figures S9.** The cancer dependence map detected by CES, CRISPR, and shRNA scores in combination.

**
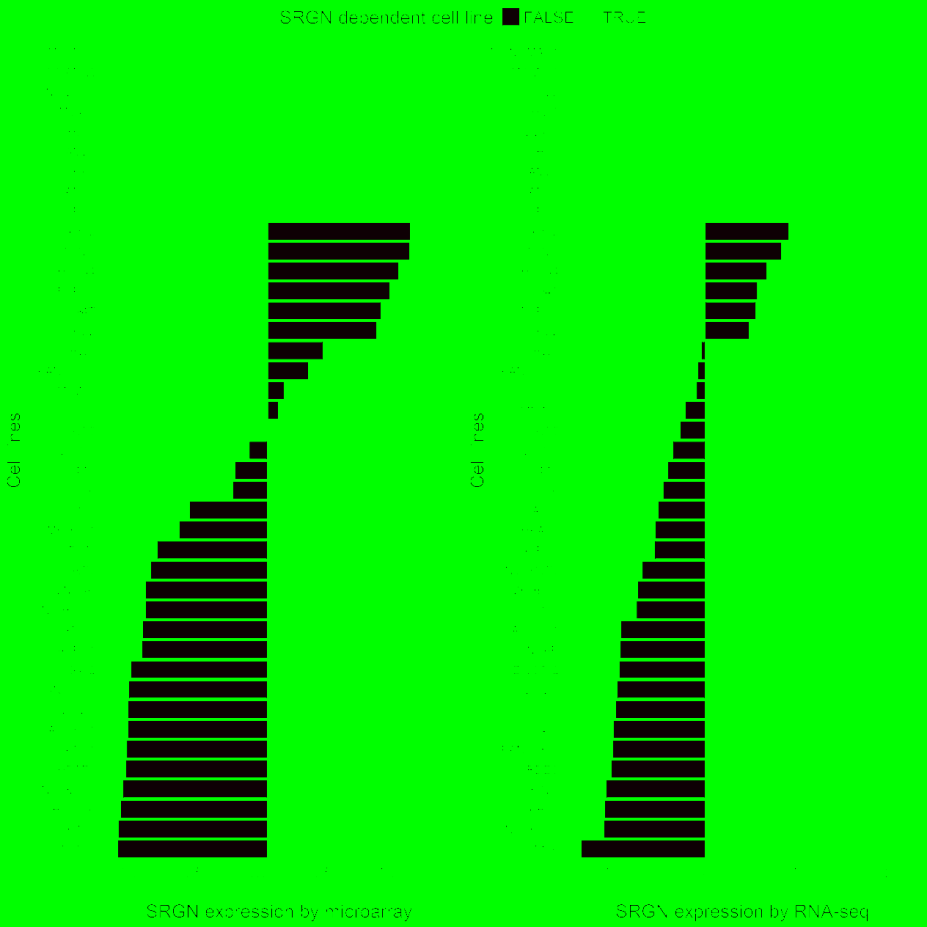
**

**Figure S10.** Gene expression of *SRGN*. *SRGN*-dependent cell lines identified by CES are highlighted.
