# Supplementary figures and images for: Corrigendum to ‘Combined gene essentiality scoring improves the prediction of cancer dependency maps’ [EBioMedicine 50 (2019) 66–79]
Source: eBioMedicine. 2020 Jan 3;51:102594. doi: 10.1016/j.ebiom.2019.12.003 (PMC6948202; doi:10.1016/j.ebiom.2019.12.003)

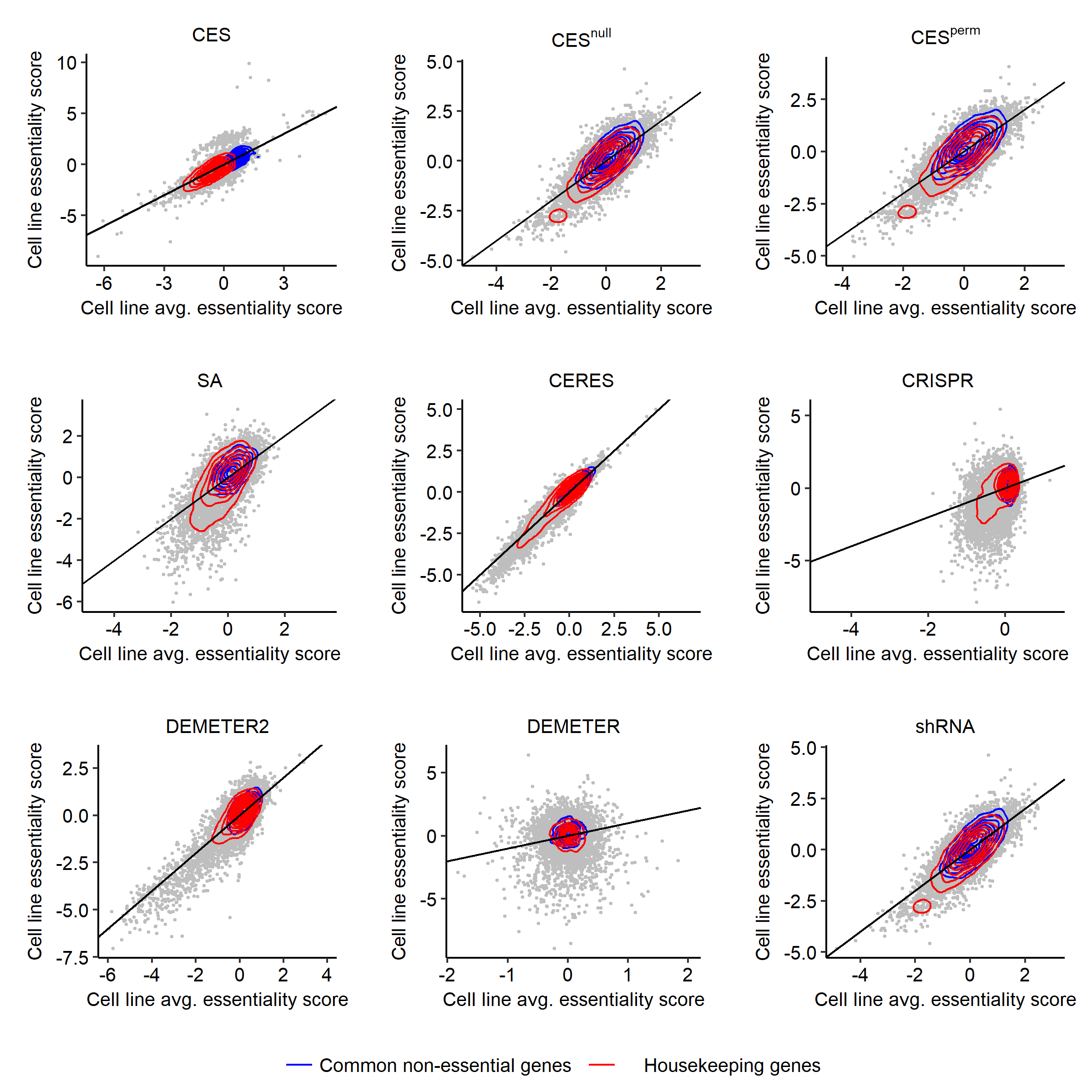


Figure S5


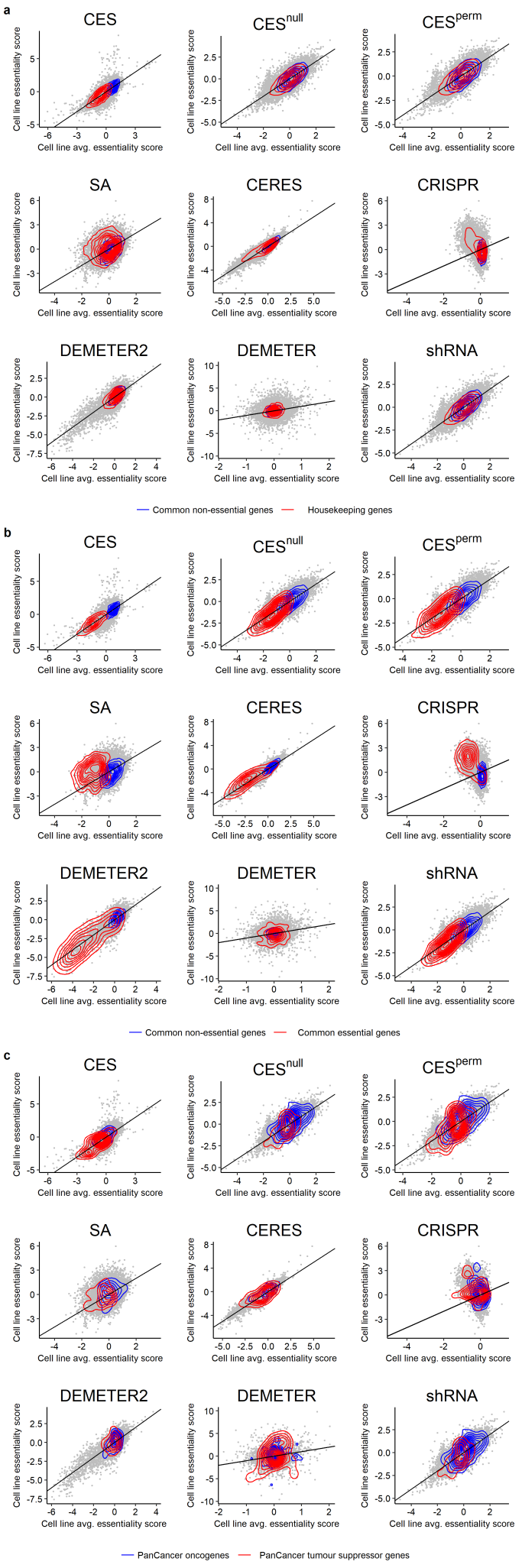


Figure S6

Supplement: Supplementary file 1 [file mmc1.docx]
